# Supplementary material for: Innate immune responses against the fungal pathogen Candida auris
Source: Nat Commun. 2022 Jun 21;13:3553. doi: 10.1038/s41467-022-31201-x (PMC9213489; doi:10.1038/s41467-022-31201-x)
Supplement: Supplementary file 1 — Supplementary Information [file 41467_2022_31201_MOESM1_ESM.pdf]

Supplementary Information

Innate immune responses against the fungal pathogen *Candida auris*

Yuanyuan Wang[1,3,4#], Yun Zou[1,3,4#], Xiaoqing Chen[1,3], Hao Li [5], Zhe Yin[2], Baocai Zhang[6], Yongbin Xu[7], Yiquan Zhang[8], Rulin Zhang[9], Xinhua Huang[1], Wenhui Yang[2], Chaoyue Xu[1,4,10], Tong Jiang[1,3], Qinyu Tang[11], Zili Zhou[1], Ying Ji[1,3], Yingqi Liu[12], Lingfei Hu[2], Jia Zhou[13], Yao Zhou[14], Jingjun Zhao[11], Ningning Liu[13], Guanghua Huang[15], Haishuang Chang[16], Wenxia Fang[14], Changbin Chen[1,4]*, Dongsheng Zhou[2]*

[#] Contributed equally

* Corresponding authors.

E-mail: cbchen@ips.ac.cn; dongshengzhou1977@gmail.com

Supplementary Figures and Figure legends

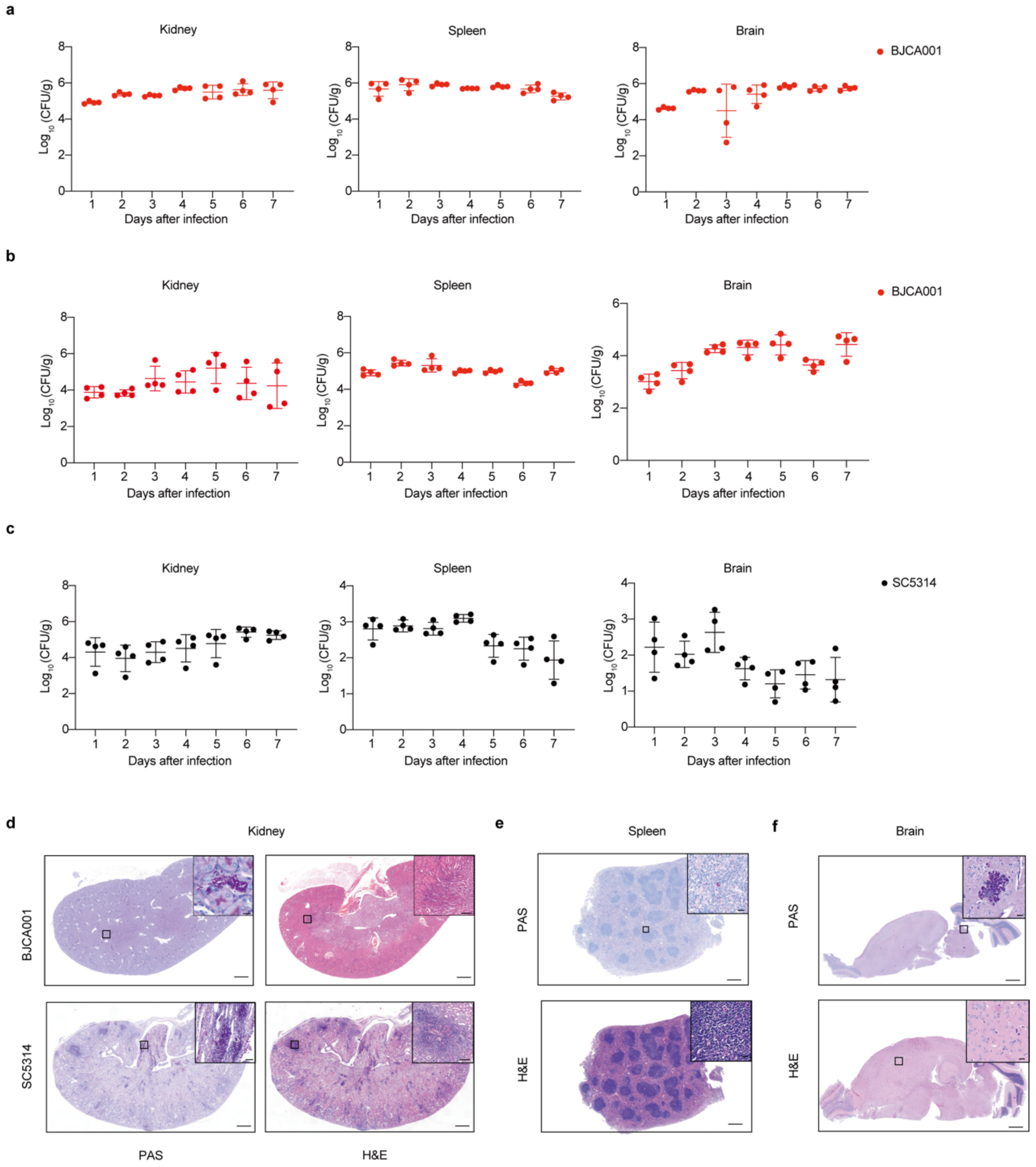

Supplementary Fig.1: High levels of *C. auris* cells are persistently detected from different organs of immunocompetent mice during systematic infections. a and **b** C57BL/6 mice were infected via the tail vein with viable *C. auris* yeasts (n=4 mice/group/time point). Total kidney, spleen and brain tissues of individual mice were analyzed in triplicate for outgrowth of *C. auris* at different time points. **(a)** 2×10^7

CFU/mice, **(b)** 1×10^6 CFU/mice. **c** For comparison, a parallel examination of tissue fungal burden in *C. albicans* was also undertaken, using 5×10^4 viable yeasts (n=4 mice/group/time point). **d** Representative histopathology cross-sections of kidneys from *C. auris*- or *C. albicans*- infected mice were stained with PAS (Scale bar = 100 μm, insets) or H&E (Scale bar = 10 μm, insets) at day 4 post-infection. **e-f** Representative histopathology cross-sections of spleens (**e**) and brains (**f**) from *C. auris*- infected mice were stained with PAS (Scale bar = 100 μm, insets) or H&E (Scale bar = 20 μm, insets) at day 4 post-infection. Images are representative of twelve mice from two independent experiments. Data are expressed as mean ± SD and are representative of two independent experiments. Source data are provided as a Source Data file.

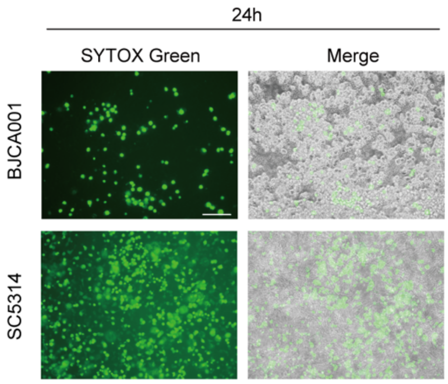

Supplementary Fig.2: *C. albicans* caused robust macrophage cell death 24 h after co-incubation. BMDMs were co-incubated with *C. auris* BJCA001 or *C. albicans* SC5314 at a MOI of 5 for 24h, and samples were stained by membrane-impermeable fluorescent dyes SYTOX green and the nonviable cells (green) were viewed by fluorescent microscopy (Olympus IX73), Scale bar = 10 μm. Images are representative of two independent experiments.

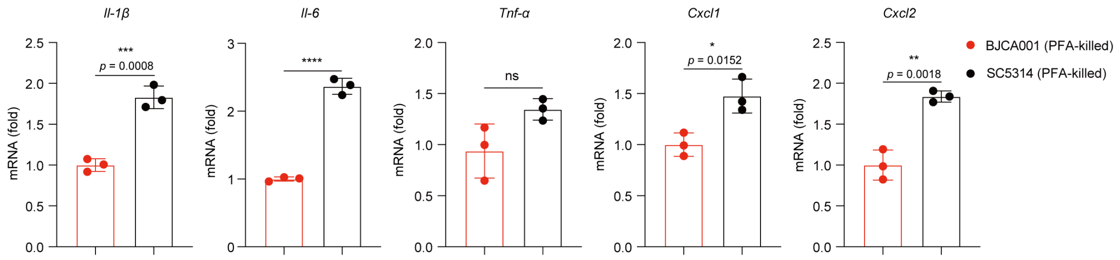

Supplementary Fig.3: Compared to PFA-fixed *C. auris* cells, *C. albicans* yeasts treated similarly induced higher levels of cytokine/chemokine gene expression during coculture with BMDMs. Expression levels of IL-1β, IL-6, TNF-α, CXCL1 and CXCL2, as determined by real-time RT-qPCR, in BMDMs that were infected with PFA-killed form of *C. auris or C. albicans* for 3 h (n=3). Results were normalized to the expression of the control gene GAPDH and are presented relative to those of *C. auris* cells, set as 1. Data are expressed as mean ± SD and are representative of three independent experiments. ns, no significance; $*p < 0.05$, $***p < 0.001$, $****p < 0.0001$, by two-side unpaired *t*-test. Source data are provided as a Source Data file.

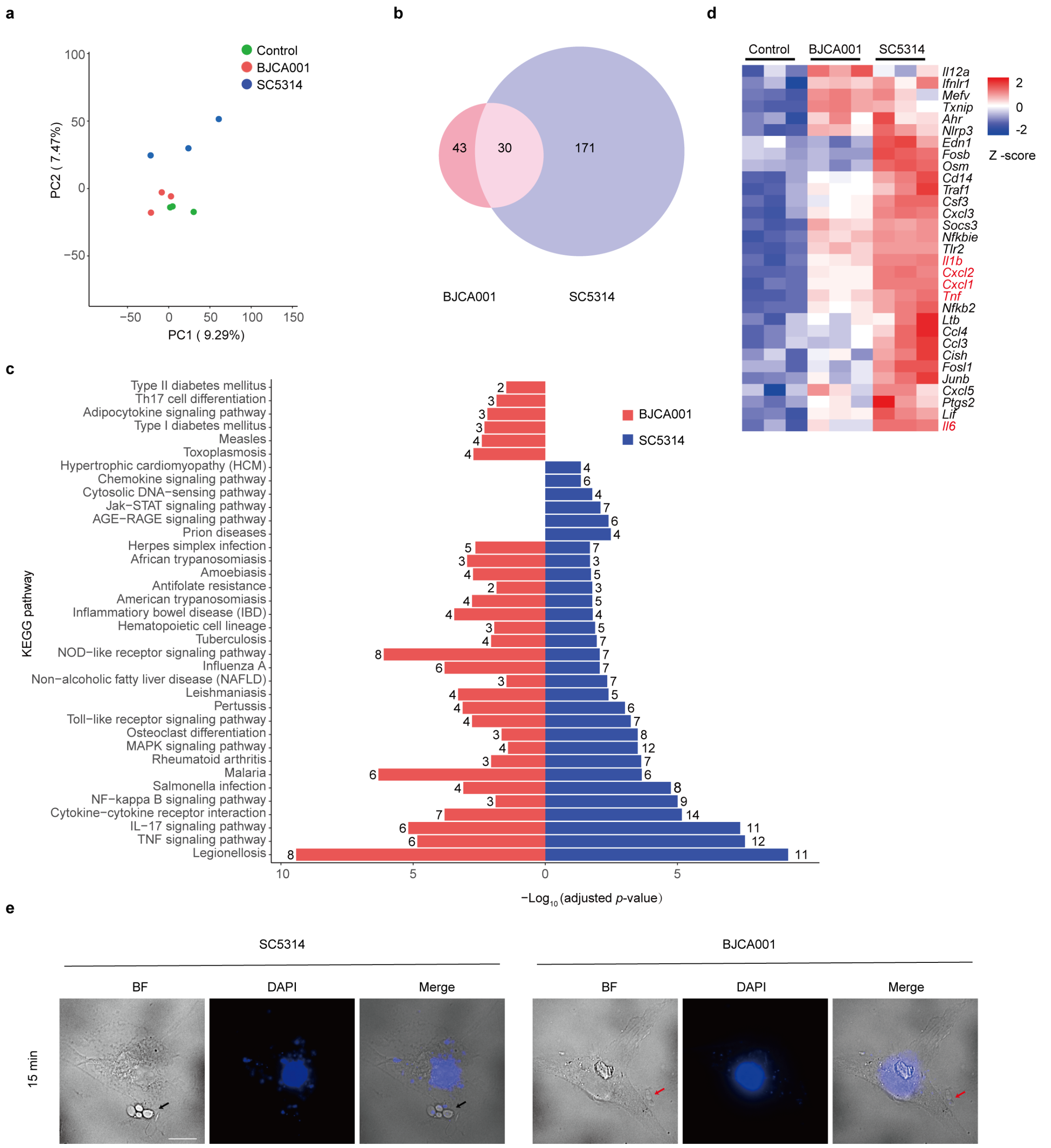

Supplementary Fig.4: *C. auris* potently inhibits early phase innate immune response during the interaction with BMDMs. a Principal component analysis (PCA) of the normalized RNA-seq data of BMDMs in response to challenge with PBS (control), live *C. auris* or *C. albicans* yeast cells (MOI=5) for 3 h. **b** Venn diagram showing differentially expressed genes (DEGs; fold change ≥ 2, adjusted $p \leq 0.05$). **c** A schematic diagram of a bar chart for the top 30 enriched KEGG pathways ranked

according to their values of $-\log_{10}$ (adjusted *p*-value). The number of DEGs in a given pathway is listed. The *p*-value was calculated based on the cumulative hypergeometric distribution and adjusted using Benjamini-Hochberg correction for multiple comparisons. **d** Heatmap of changes in gene expression of selected innate immune-related genes. Genes encoding pro-inflammatory cytokines and chemokines who were further analyzed in the study were highlighted in red color. **e** The yeast-form morphology of live *C. albicans* or *C. auris* cells during coculture with BMDMs for 15 min, respectively. Images are captured by fluorescent microscopy and processed in the ImageJ, Scale bar = 5 μm. Images are representative of two independent experiments.

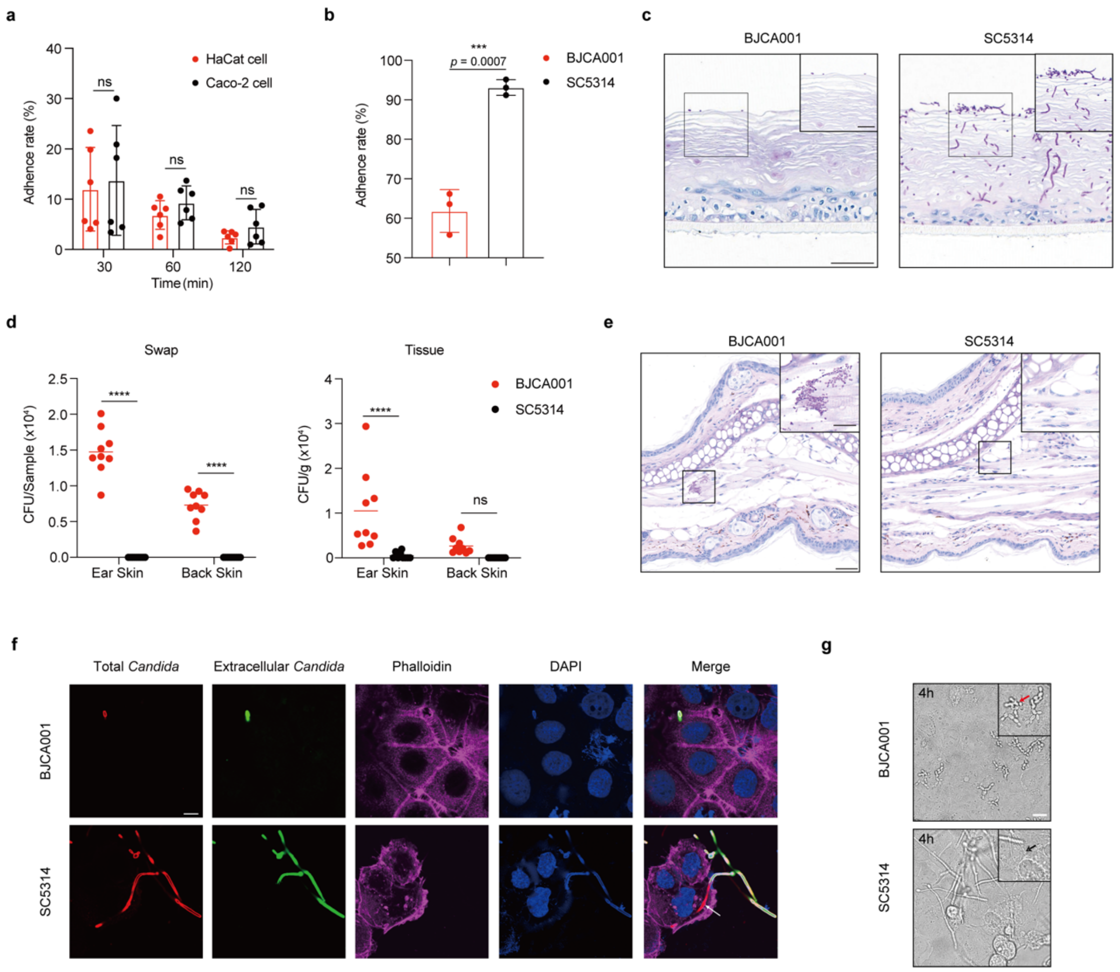

Supplementary Fig.5: *In vitro* and *in vivo* studies investigating the interaction of *C. auris* or *C. albicans* cells with the skin. a Time course of adherence to HaCat or Caco-2 cells by *C. auris* BJCA001. Adherence of *Candida* species to both cells was determined by counting the adhering CFU of the yeast and expressed as the percentage of original inoculum (n=6). **b** Adhesion of a reconstructed human epidermis (RHE) with *C. auris* BJCA001 or *C. albicans* SC5314 for 24 h at 37 ℃ (n=3). Adherence of *Candida* species to RHE was determined using the same method as in a. **c** Representative histopathological cross-sections of RHE 24 h after infection with *C. auris* or *C. albicans*. (Scale bar = 20 μm, insets) **d** Colonization of *C. auris* or *C.*

albicans on skin surface and tissue. Colony forming units (CFUs) per swab (left) or per gram of tissue (right) in mice (n=9) inoculated with *C. auris* BJCA001 or *C. albicans* SC5314 were plotted. **e** Representative histopathological cross-sections of ear pinna 14 d after infection with *C. auris* or *C. albicans*. (Scale bar = 20 μm, insets) **f** Confocal micrographs showing endocytosis of *C. auris* or *C. albicans* by human colorectal adenocarcinoma cell line (Caco-2). White arrow indicates that *C. albicans* hyphae were endocytosed by Caco-2. Scale bar, 5 μm. **g** Representative snapshots were taken from live cell videos after 4 h of co-incubation of *C. auris* or *C. albicans* cells with Caco-2 (MOI=5). Black arrows indicate that *C. albicans* hyphae could be endocytosed by Caco-2 cells. Scale bar, 1 μm. Data are expressed as mean ± SD and are representative of three independent experiments. ns, no significance; ***$p < 0.001$, ****$p < 0.0001$, by two-way ANOVA with Sidak's test (**a**, **d**） or two-side unpaired *t*-test (**b**). Source data are provided as a Source Data file.

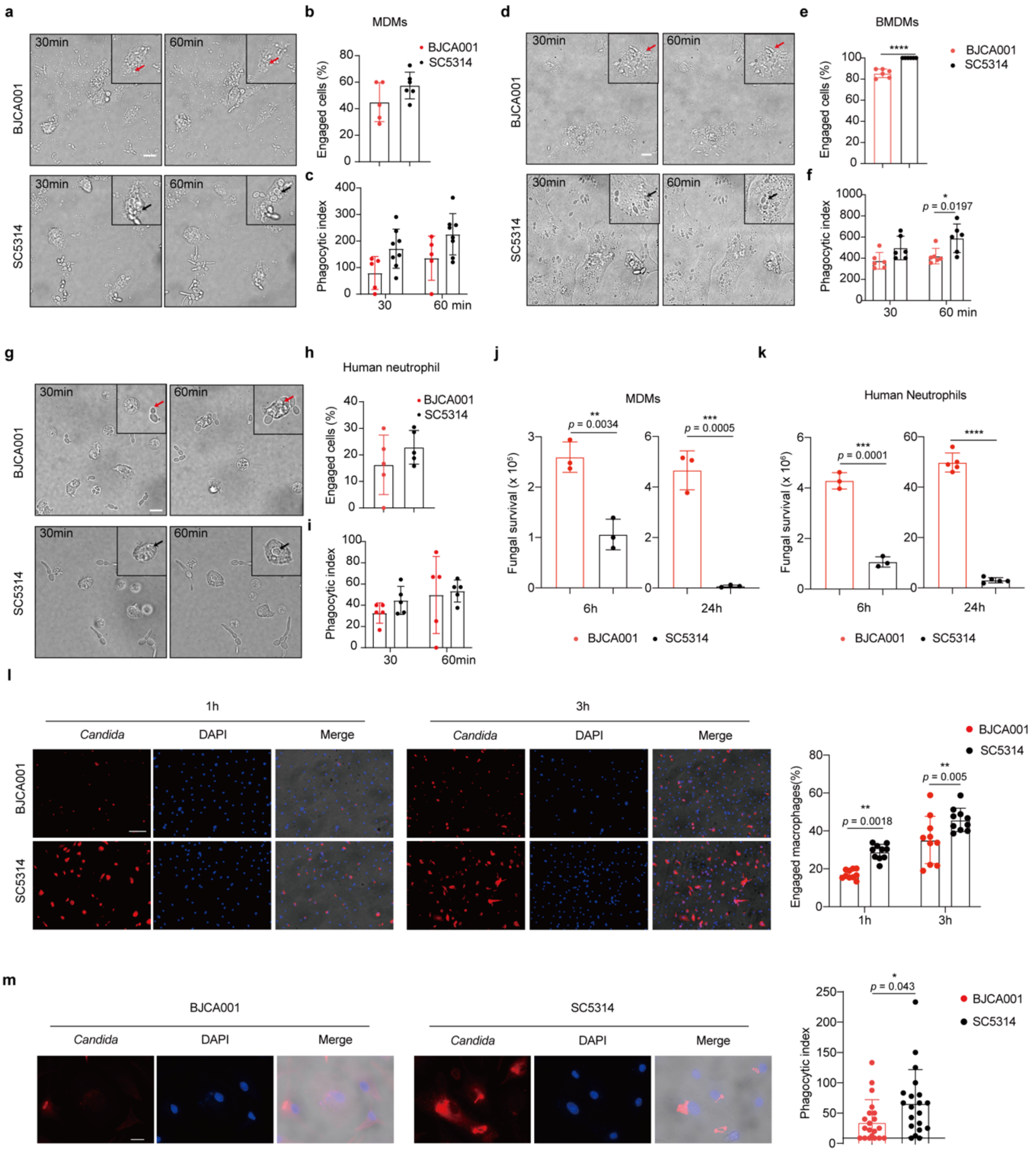

Supplementary Fig.6: Compared to *C. albicans*, *C. auris* shows a decreased ability to internalization by and damage of innate immune cells. a Representative snapshots were taken from live cell videos after 30 and 60 mins co-incubation of human MDMs with live *C. albicans* SC5314 or *C. auris* BJCA001 cells (MOI=1). Numbers in the upper left corner of each image represent the time of phagocytic events, arrows indicate the fungal cells engulfed by human MDMs (scare bar =10 μm). **b** and

c Percentage uptake and phagocytic index for human MDMs ingesting live *C. albicans* (n=5) and *C. auris* (n=8). Macrophages that have taken up at least one fungal cell were manually tracked to allow a quantitative analysis of percentage uptake during the 60 min co-incubation **(b)**. The number of fungal cells ingested (phagocytic index) per 100 macrophages was manually counted during the 30 and 60 min co-incubation **(c)**. Scare bar =10μm. **d** Representative snapshots were taken from live cell videos after co-incubation of murine BMDMs with PFA-fixed *C. albicans* SC5314 or *C. auris* BJCA001 cells (MOI=1). **e** and **f** Percentage uptake and phagocytic index for murine BMDMs ingesting formalin-fixed *C. albicans* and *C. auris* (n=6). g**-i** Live cell imaging data from co-incubations with human neutrophils and live *C. albicans* or *C. auris* (MOI=1). Data were analyzed using exactly the same methods described in **(a-c)**. Scare bar =10 μm; n=5. **j** and **k** The survival of *C. albicans* and *C. auris* after infection of human MDMs **(j)** or neutrophils **(k)** at a MOI of 1 for 6 and 24 h was evaluated by plating dilutions of sample on YPD agar (n=3). **l** *C. auris* or *C. albicans* were cocultured with BMDMs at a MOI of 5 for 1 h or 3 h, and the fungal cells were labeled by *Candida* antibody (red) and the BMDM cells were labeled by DAPI (blue). Images are captured by fluorescent microscopy and processed in the ImageJ. Macrophages that have taken up at least one fungal cells were counted by the count function of ImageJ, to allow a quantitative analysis of percentage uptake. Scare bar =10 μm; n=10. **m** The number of fungal cells ingested (phagocytic index) per 100 macrophages was manually counted during 60 min co-incubation, using the enlarged image of **l**. Scare bar =5 μm; n=20. Data are

expressed as mean ± SD and are representative of three independent experiments. ns, no significance; *$p < 0.05$, **$p < 0.01$, ***$p < 0.001$, ****$p < 0.0001$, by two-way ANOVA with Sidak's test (**c**, **f**, **i, l**） or two-side unpaired t-test (**b**, **e**, **j, k, m**). Source data are provided as a Source Data file.

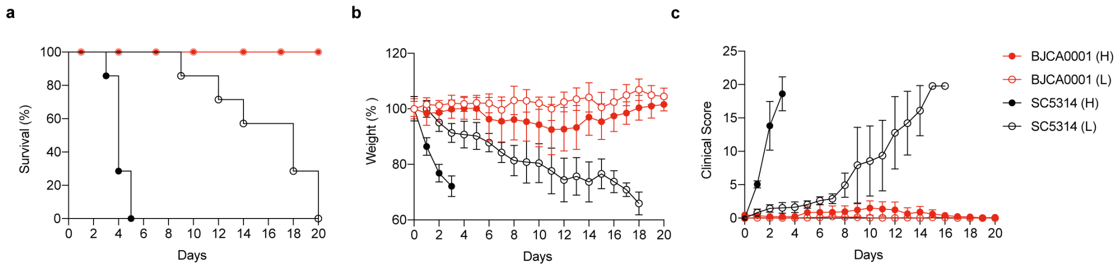

Supplementary Fig.7 The inoculation size significantly impacts the virulence of *C. albicans* in mice. Groups of C57BL/6 mice (n=7) were infected intravenously with *C. auris* BJCA001 (H: 2×10^{7} cells/mouse; L: 1×10^{6} cells/mouse) or *C. albicans* SC5314 (H: 1×10^{6} cells/mouse; L: 5×10^{4} cells/mouse). **a** Shown were the survival percentages. **b** Weight changes of infected mice during experiments. **c** Clinical scores. Data are expressed as mean ± SD and are representative of two independent experiments. Source data are provided as a Source Data file.

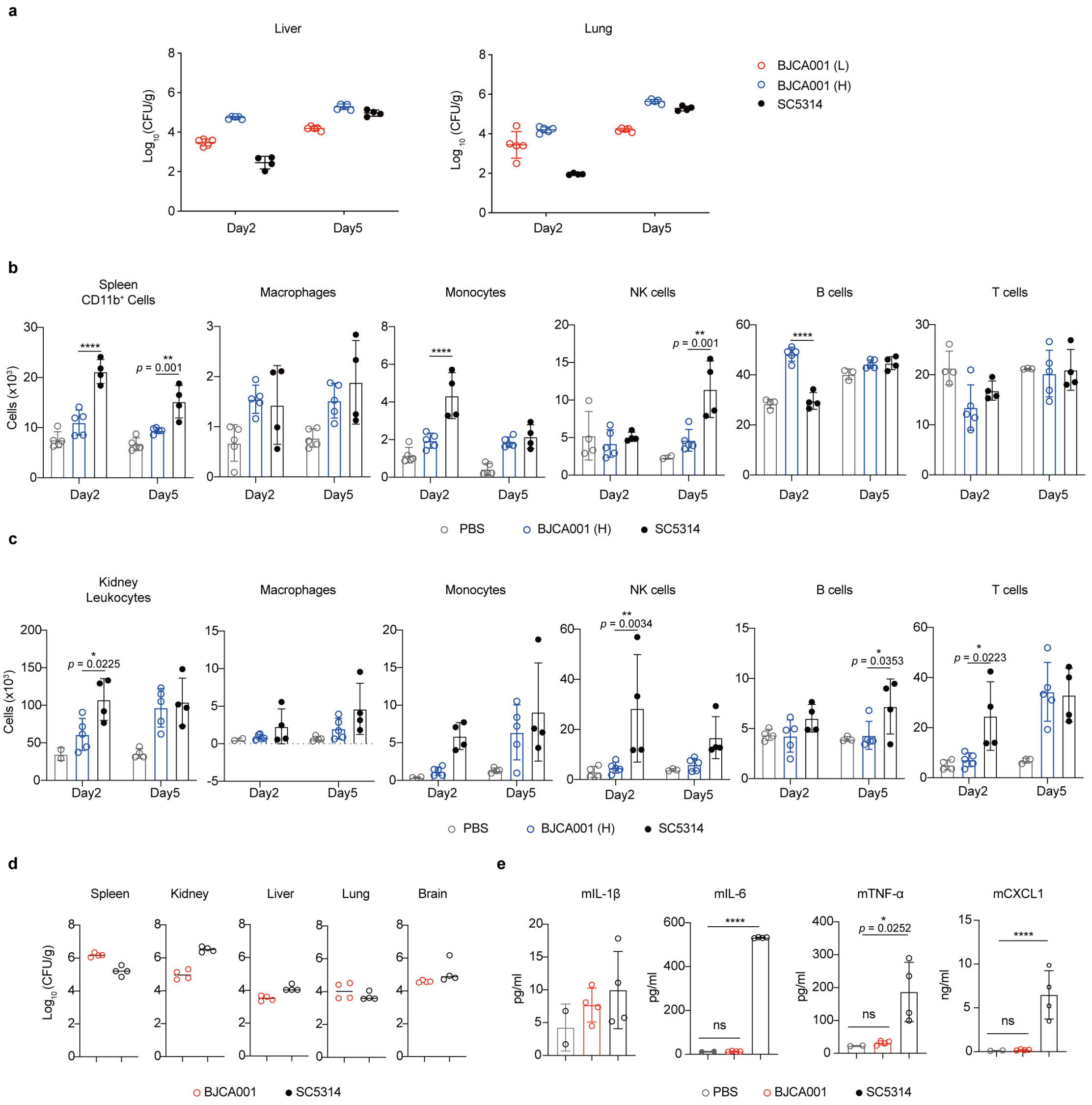

Supplementary Fig.8: *In vivo* evidence supporting the innate immune evasion of *C. auris*. a The mice (n=5 mice/group/time point) were intravenously infected with either 1 × 10^6 (L) or 2 × 10^7 (H) of *C. auris* yeast cells and tissue fungal burdens were measured at day 2 and 5 post-inoculations. For comparison, a parallel examination of tissue fungal burden was also undertaken using 5x10^4 viable *C. albicans* yeasts (n=4 mice/group/time point). **b** and **c** Shown are the major immune cell populations in spleen **(b)** and kidney **(c)** cell suspensions of mice that were infected without (PBS, negative control) or with live *C. auris* (n=5) or *C. albicans* (n=4) for 2 and 5 days. The number

of each immune population was determined by flow cytometry. *C. albicans* was injected into mice at an inoculum of 5×10^4 CFU and *C. auris* was injected at an inoculum of 2×10^7 CFU(H). **d** Quantitative fungal burdens in indicated tissues of C57BL/6 mice (n=4 mice/group/time point) intravenously infected with *C. albicans* or *C. auris* (1×10^6 CFU/mice) at day 2 and 5 post-inoculations. **e** Serum cytokine and chemokine levels, as determined by ELISA, in mice treated as in **(d)**. Data are expressed as mean ± SD and are representative of three independent experiments. ns, no significance; *$p < 0.05$; **$p < 0.01$; ****$p < 0.0001$; by two-way ANOVA with Tukey's test (**b**, **c**) or one-way ANOVA with Dunnett's test (**e**). Source data are provided as a Source Data file.

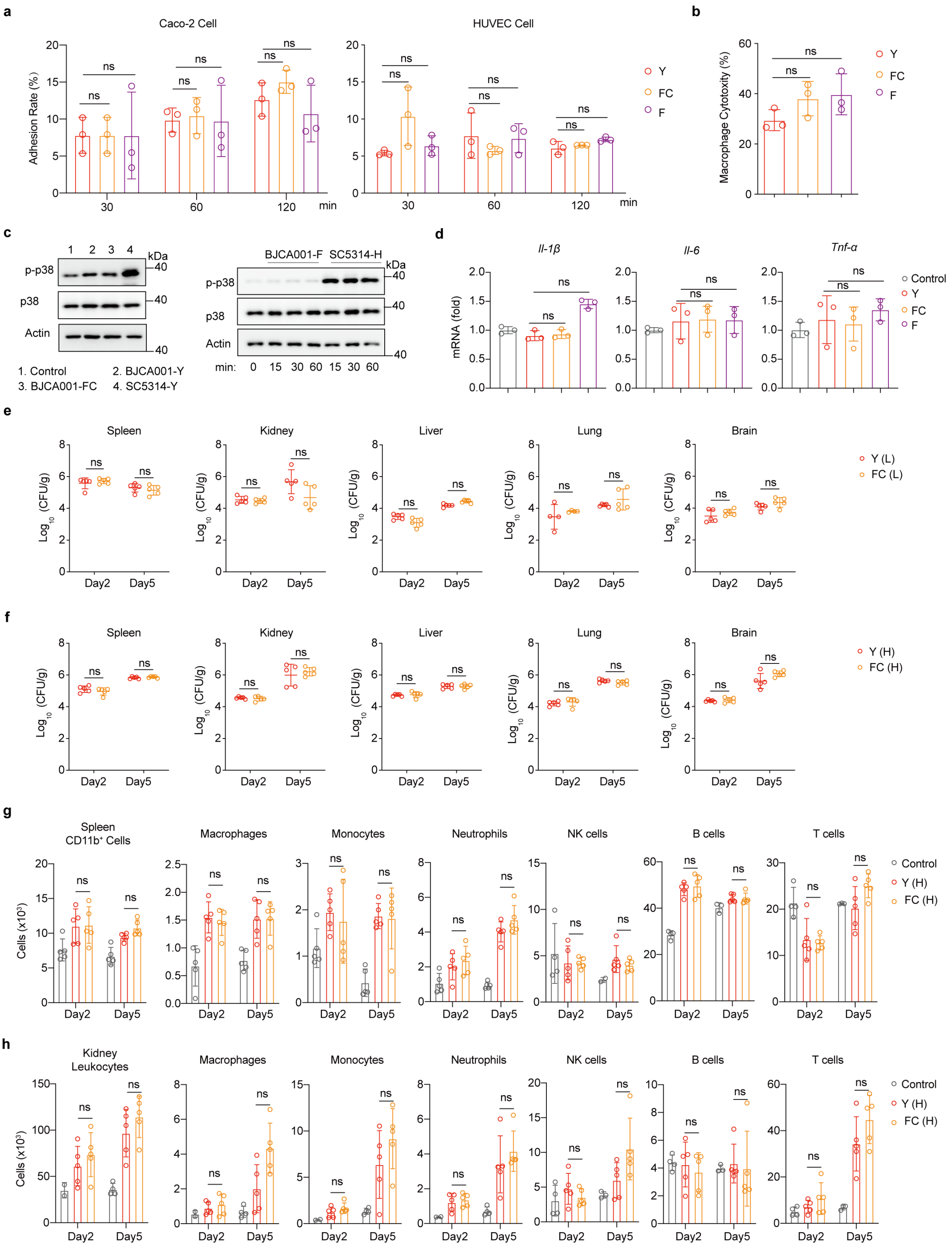

Supplementary Fig.9: The different morphological forms of *C. auris* show no changes of adhesion by epithelial and endothelial cells, macrophage damage, p38 MAPK activation, tissue fungal burns and immune cell populations in different host organs. a Time course of adherence to Caco-2 or HUVEC cells by the

yeast (Y), filamentation-competent (FC) yeast or filamentous (F) form of *C. auris*. Adherence to epithelial or endothelial cells was determined by counting the adhering CFU of the yeast and expressed as the percentage of the original inoculum (n=3). **b** Yeast, FC and filament forms of *C. auris* show similar damage to BMDMs (n=3). **c** Immunoblot analysis for detection of p38 activation. Upper panel, BMDMs were stimulated without (PBS control) or with the yeast (Y) or filament-competent (FC) form of *C. auris* or *C. albicans* yeast cells at MOI=5 for 15 min; Bottom panel, BMDMs were stimulated with filamentous form of *C. auris* (F) or hyphal form of *C. albicans* (H) at MOI=5 for the indicated amounts of time. Cell lysates were subjected to immunoblots with the indicated p38 MAPK antibodies. **d** Expression levels of IL-1β, IL-6 and TNF-α, as determined by real-time RT-qPCR, in BMDMs that were infected without (PBS control) or with the yeast (Y), filamentation-competent (FC) yeast or filamentous (F) form of *C. auris* (MOI=5) for 3 h (n=3). Results were normalized to the expression of the control gene GAPDH and are presented relative to those of negative control, set as 1. **e** and **f** Quantitative fungal burdens of spleen, kidney, liver, lung and brain tissues of C57BL/6 mice (n=5 mice/group/time point) intravenously infected with either 1×10^6 CFU **(e)** or 2×10^7 CFU **(f)** of *C. auris* in a yeast (Y) or filament-competent (FC) form at day 2 and 5 post-inoculations. **g** and **h** Shown are the major immune cell populations in spleen **(g)** and kidney **(h)** cell suspensions of mice that were infected without (PBS, negative control) or with the yeast (Y) or filamentation-competent (FC) yeast form of *C. auris* BJCA001 for 2 and 5 days. *C. auris* was injected into mice at an inoculum of

2×10^7 CFU (H). Data are expressed as mean ± SD and are representative of three independent experiments. ns, no significance; by two-way ANOVA with Tukey's test (**a**, **e-h**) or one-way ANOVA with Dunnett's test (**b, d**). Source data are provided as a Source Data file.

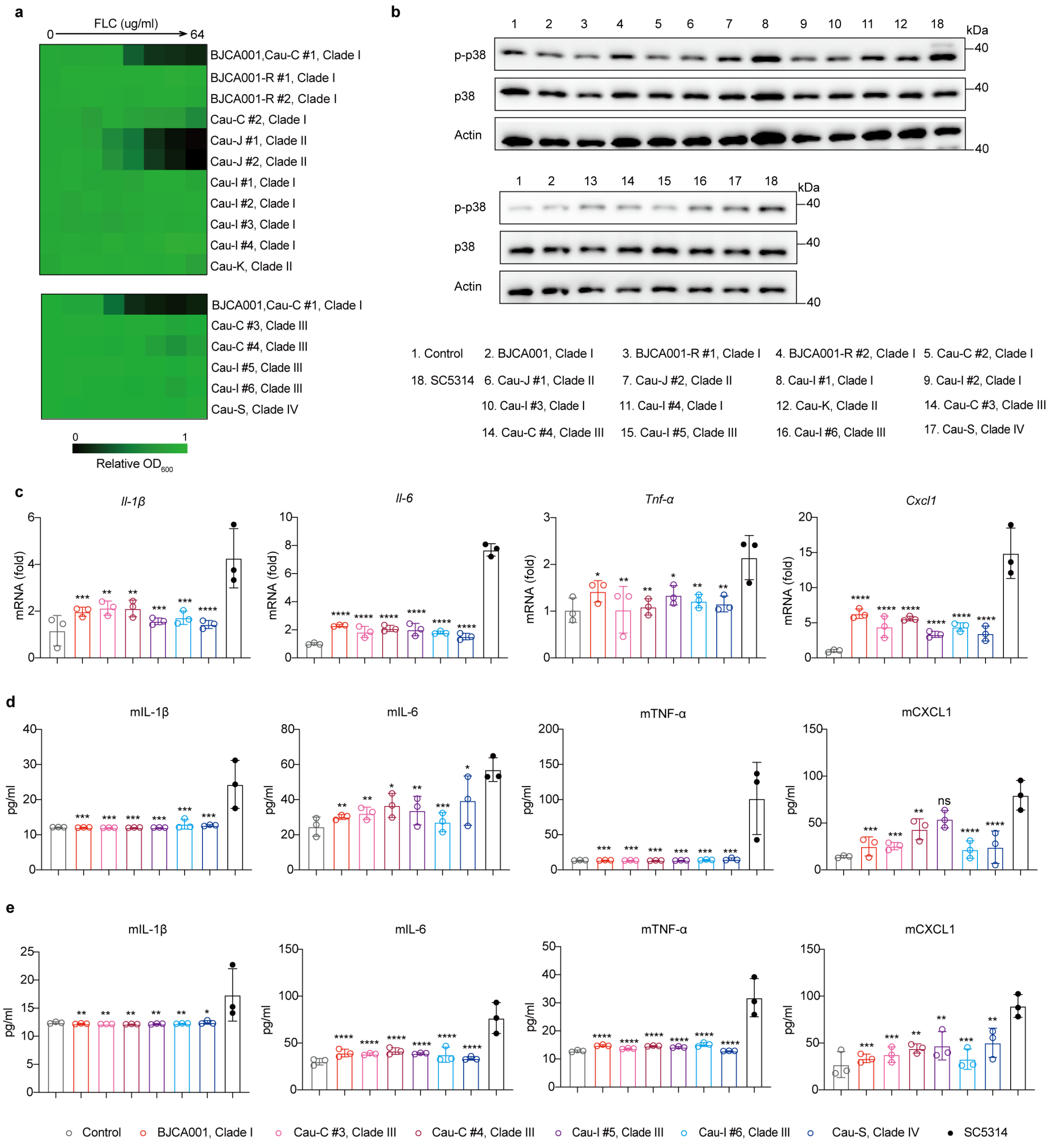

Supplementary Fig.10: Stimulation of BMDMs with the eleven fluconazole-resistant isolates of *C. auris* showed conserved features of host innate immune response. a Heatmap of fluconazole resistance in each of the 16 *C. auris* isolates, as measured by the checkerboard assay. **b** Immunoblot analysis for detection of the indicated p38 MAPKs, using lysates from BMDMs that were stimulated with each of 16 *C. auris* clinical isolates at MOI=5 for 15 min. **c-e** The innate immune response against various fluconazole-resistant clinical isolates of *C. auris*. **(c)** Expression of IL-1β, IL-6,

TNF-α and CXCL1 were analyzed by real-time RT-qPCR. BMDMs were stimulated without (PBS control) or with each of the five fluconazole-resistant clinical isolates of *C. auris* or *C. albicans* SC5314 at MOI=5 for 3 h. Results were normalized to the expression of the control gene GAPDH and are presented relative to those of negative control, set as 1. (n=3; from left to right, IL-1β: p=0.0007, 0.0013, 0.0012, 0.0001, 0.0002; TNF-α: p=0.0469, 0.0018, 0.003, 0.024, 0.0084, 0.0051). **(d)** Production of cytokines and chemokines were analyzed by ELISA after BMDMs were stimulated with indicated strains at MOI=5 for 6 h. (n=3; from left to right, IL-1β: p=0.0001, 0.0001, 0.0001, 0.0001, 0.0003, 0.0002; IL-6: p=0.0021, 0.0043, 0.0195, 0.0073, 0.0008, 0.0491; TNF-α: p=0.0001, 0.0001, 0.0001, 0.0001, 0.0002, 0.0002; CXCL1: p=0.0001, 0.0001, 0.0054). **(e)** Similar to **d**, except for lower inoculum of *C. auris* (MOI=0.1) and longer stimulation time (24 h). (n=3; from left to right, IL-1β: p= 0.0089, 0.0082, 0.008, 0.0084, 0.0093, 0.0127; CXCL1: p=0.0001, 0.0003, 0.001, 0.0019, 0.0001, 0.0038). Data are expressed as mean ± SD and are representative of three independent experiments. ns, no significance; *p < 0.05; **p < 0.01; ***p < 0.001; ****p < 0.0001; by two-way ANOVA with Tukey's test (**c-e**). Source data are provided as a Source Data file.

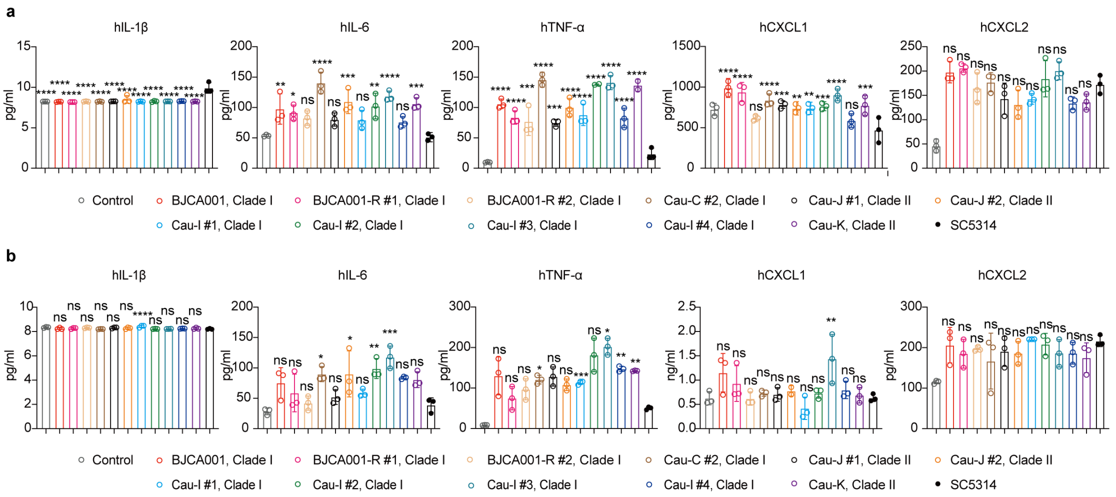

Supplementary Fig.11: Pro-inflammatory response by human PBMCs upon interaction with *C. albicans* and *C. auris*. a Production of the cytokines and chemokines were analyzed by ELISA after human PBMCs were stimulated with indicated strains at a MOI of 5 for 6 h. (n=3; from left to right, hIL-6: *p*=0.0036, 0.0131, 0.0002, 0.0012, 0.0005; hTNF-α: *p*=0.0001, 0.0001, 0.0002; hCXCL1: *p*=0.0003, 0.0019, 0.0018, 0.0007, 0.0004). **b** Production of the cytokines and chemokines were analyzed by ELISA after human PBMCs were stimulated with indicated strains at a MOI of 0.1 for 24 h. (n=3; from left to right, hIL-6: *p*=0.0205, 0.0194, 0.0047, 0.0002; hTNF-α: *p*=0.0334, 0.0004, 0.0254, 0.0011, 0.0021; hCXCL1: *p*= 0.0032). Data are expressed as mean ± SD and are representative of three independent experiments. ns, no significance; **p* < 0.05; ***p* < 0.01; ****p* < 0.001; *****p* < 0.0001; by one-way ANOVA with Sidak's test (**a**, **b**). Source data are provided as a Source Data file.

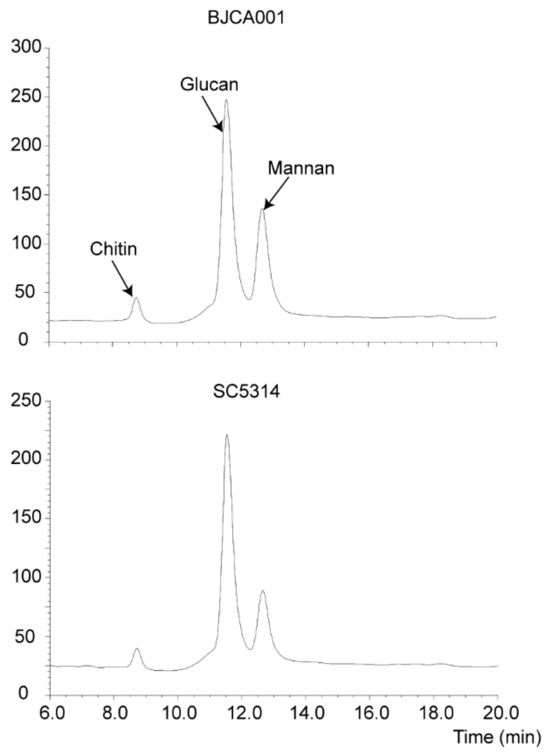

Supplementary Fig.12: The components and size of *C. auris* and *C. albicans* cell wall. The HPLC chromatogram of the cell wall components of *C. auris* BJCA001 (upper) and *C. albicans* SC5314 (bottom).

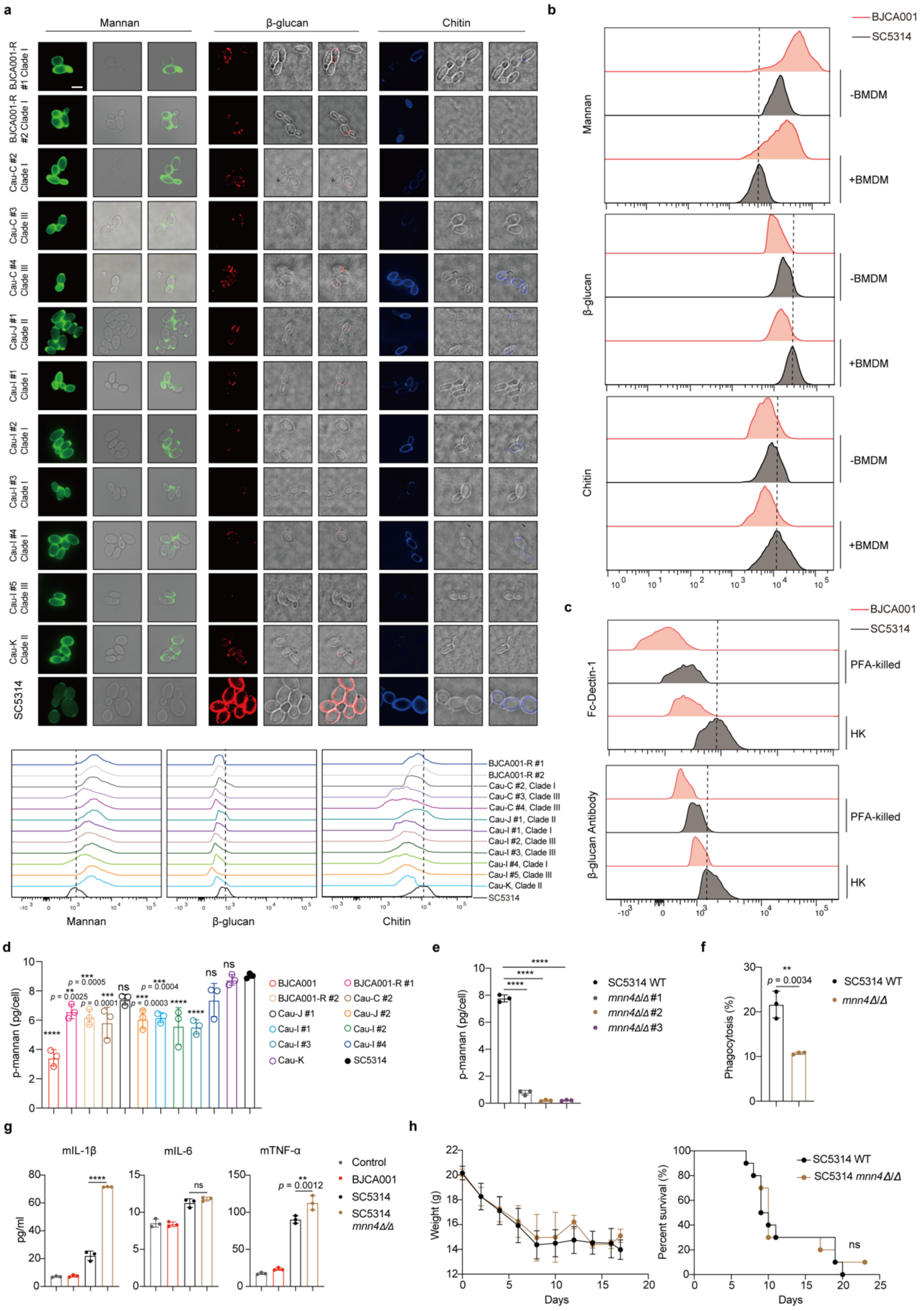

Supplementary Fig.13: The cell wall phosphomannan of *Candida* species is important for phagocytosis but not for cytokine induction and virulence. a

Representative fluorescence micrographs showing the three main polysaccharide layers of *C. auris* or *C. albicans* cell wall. Exponentially growing fungal cells were stained with ConA-FITC to visualize mannan, Fc-Dectin-1 to visualize β-glucan, and calcofluor white (CFW) to visualize chitin. Scale bar, 5 μm. The intensity of fluorescence representing the level of mannan, β-glucan or chitin (upper) and was quantified by flow cytometry (bottom). Data are representative of three independent and reproducible experiments. **b** Distribution pattern of different cell wall components (mannan, β-glucan and chitin) in *C. albicans* and *C. auris* cells during co-incubation without or with BMDMs. Cells of the indicated strains were co-incubated without or with BMDMs at a MOI of 1 for 1 h and then fixed. After staining, exposure of cell wall mannan (ConA), β-glucan (Fc-Dectin-1) and chitin (CFW) was examined by flow cytometry. **c** Exposure of β-glucan cell wall in PFA- or heat-killed yeast cells of *C. auris* or *C. albicans* was quantified by flow cytometry. Exponentially growing fungal cells were killed by 4% paraformaldehyde (PFA) or heat (65℃), and stained with Fc-Dectin-1 or β-glucan antibody to visualize β-glucan. **d** The results of quantitative Alcian blue binding assays. Stationary phase cells of the indicated strains were suspended in Alcian Blue and incubated at room temperature for 10 min prior to analysis in a spectrophotometer (n=3). **e** Binding of Alcian Blue to WT and *mnn4Δ/Δ* mutant of *C. albicans* (n=3). **f** BMDMs were infected with *C. albicans* WT or *mnn4Δ/Δ* at a MOI of 1 for 1 h. The phagocytized fungal cells were collected, diluted and plated on YPD agar (n=3). **g** Production of IL-1β, IL-6 and TNF-α, as determined by ELISA, in the culture

supernatants of BMDMs that were infected without or with *C. albicans* WT or *mnn4Δ/Δ* cells (MOI=5) for 6 h (n=3). **h** Groups of C57BL/6 mice (n=10, 1 × 10^5 cells/mouse) were infected intravenously with *C. albicans* WT or *mnn4Δ/Δ* cells, and the weight changes (left) and survival percentages (right) were shown. Data are expressed as mean ± SD and are representative of three independent experiments. ns, no significance; ***p* < 0.01; ****p* < 0.001; *****p* < 0.0001, by one-way ANOVA with Dunnett's test (**d, e**) or Sidak's test (**g**), two-side unpaired *t*-test (**f**) or Log-rank test (**h**). Source data are provided as a Source Data file.

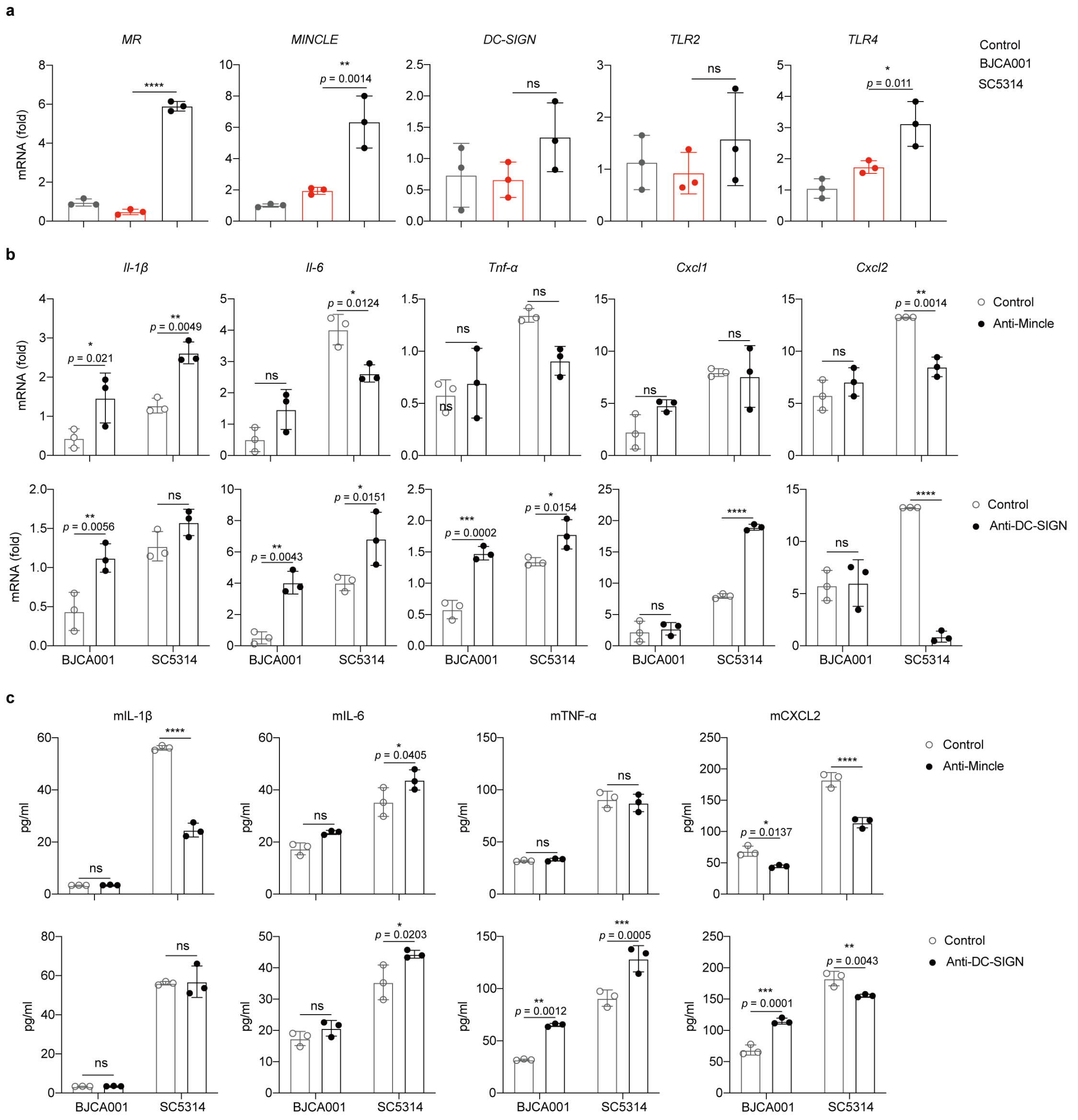

Supplementary Fig.14: The involvement of host mannan receptors in *Candida*-macrophage interaction. a Relative expression levels of genes encoded by mannan-related receptors, as determined by RT-qPCR, in BMDMs that were infected with *C. auris* or *C. albicans* (MOI=5) for 3h (n=3). Results were normalized to the expression of the control gene GAPDH and are presented relative to those of negative control, set as 1. **b** BMDMs were incubated with live *C. auris* or *C. albicans* yeast cells (MOI=5) after 1 h pre-incubation with anti-mincle (0.6 μg/ml) or anti-DC-SIGN (1 μg/ml). After 3

h incubation at 37℃, Relative expression levels of IL-1β, IL-6, TNF-α, CXCL1 and CXCL2 were determined by real-time RT-qPCR (n=3). Results were normalized to the expression of the control gene GAPDH and are presented relative to those of negative control. **c** BMDMs were treated as in **b**. After 6 h incubation at 37℃, production of IL-1β, IL-6, TNF-α and CXCL2 in the culture supernatants of BMDMs was determined by ELISA (n=3). Data are expressed as mean ± SD and are representative of three independent experiments. ns, no significance; *$p < 0.05$; **$p < 0.01$; ***$p < 0.001$, ****$p < 0.0001$; by one-way ANOVA with Sidak's test (**a**) or two-way ANOVA with Sidak's test (**b**, **c**). Source data are provided as a Source Data file.

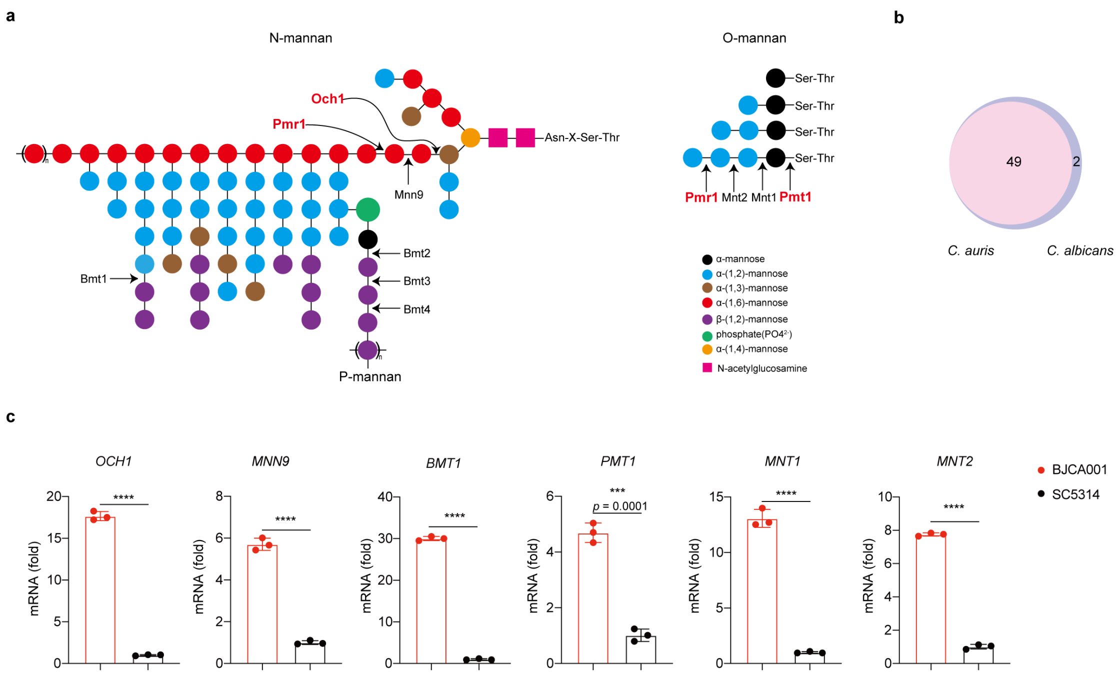

Supplementary Fig.15: The family of fungal-specific mannosyltransferases. a The coloring schematic diagram shows the enzymatic sites of different transferase enzymes involved in the linkage or addition of mannose residues in side chains of the molecule and the primary structure of mannan in *Candida* species (adapted from Murciano *et al*., 2011). Three enzymes (Pmr1, Pmt1 and Och1) are marked in red and the corresponding homologous genes were used to generate gene deletion mutants in the *C. auris* BJCA001 strain background and applied in this study. **b** Venn diagram showing the number of mannosyltransferases-encoding genes in *C. albicans* and *C. auris* genomes. **c** Relative expression levels of genes encoding the major protein *O*- and *N*- mannosyltransferases, as determined by RT-qPCR, in *C. auris* BJCA001 and *C. albicans* SC5314 (n=3). Results were normalized to the expression of the control gene ACTIN and are presented relative to those of SC5314, set as 1. Data are expressed as mean ± SD and are representative of three independent experiments.

***$p < 0.001$; ****$p < 0.0001$, by two-side unpaired *t*-test (**c**). Source data are provided as a Source Data file.

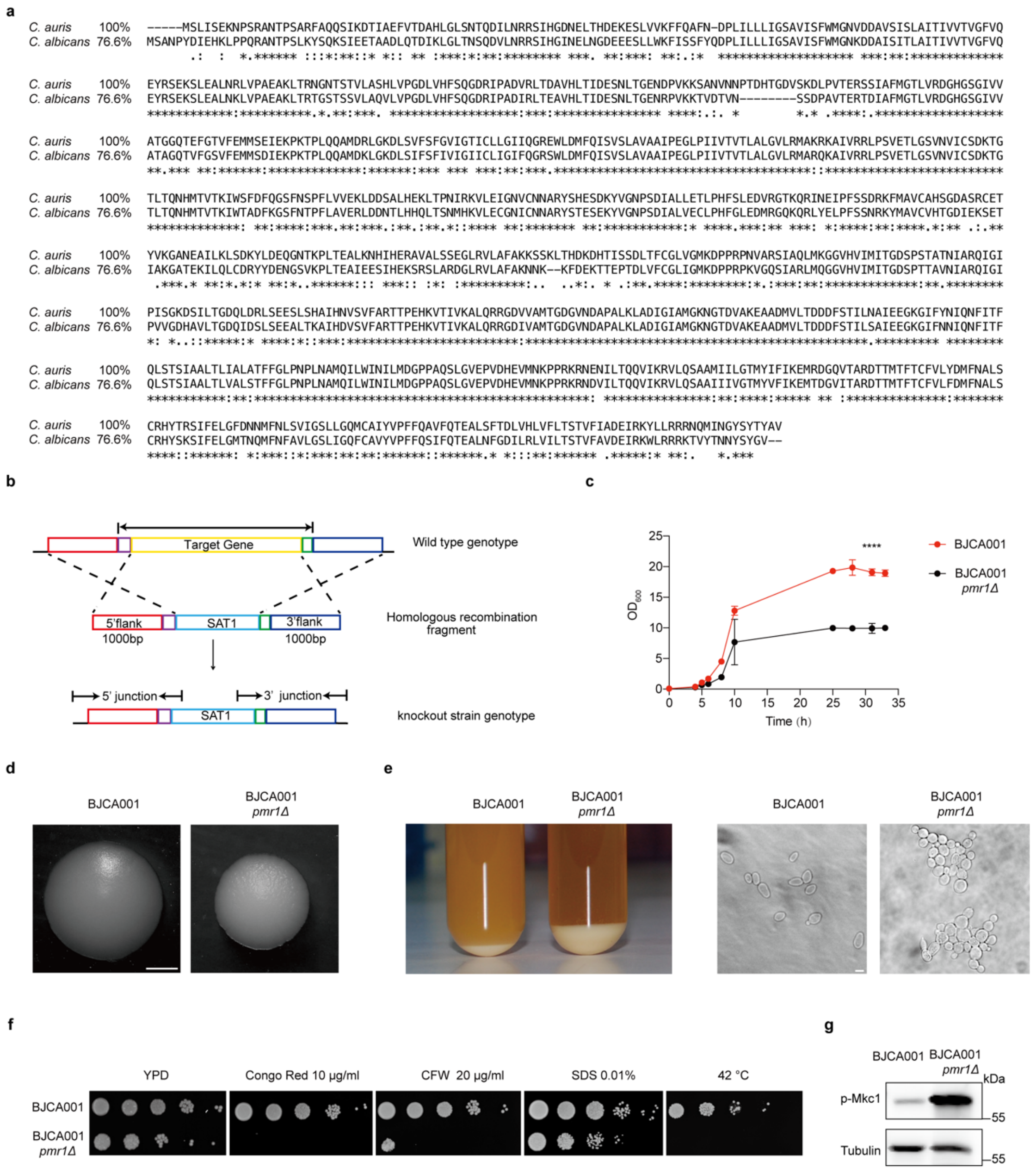

Supplementary Fig.16: The *PMR1* gene is required for cell wall integrity and cell wall composition of *C. auris*. a Alignment of deduced amino acid sequences of Pmr1 protein from *C. auris* and *C. albicans*. The homology of deduced protein sequences of Pmr1 from *C. albicans* and *C. auris* was analyzed using the ClustalW alignment tool. **b** *C. auris* gene knock out strategy through fusion PCR, using the heterologous resistance marker *SAT1*. The method of gene disruption in *C. auris* is diagrammed in

the schematic similar to that of *C. albicans*. Briefly, 5' and 3' fragments (~1kb length) flanking the ORF region of target gene, as well as the selectable marker gene cassette, are individually amplified by PCR. Following the fusion PCR reaction, a fragment (5' flank-SAT1-3' flank) was created in which the three molecules were fused and transformed to the parent strain BJCA001 by electroporation, as described in Materials and Methods. **c** Growth curve analysis of *C. auris* WT and *pmr1Δ/Δ* mutant in YPD medium at 30℃ by measuring OD_{600} at indicated time points (n=3). **d** Representative morphologies of colonies generated from *C. auris* WT or *pmr1Δ/Δ* mutant after 48 h in YPD agar. **e** *C. auris* WT and *pmr1Δ/Δ* mutant cells from overnight YPD cultures were washed, diluted, and regrown in fresh YPD medium to logarithmic growth phase at 30℃. Cultures were either photographed (left) or checked for cell morphology by light microscopy (right). **f** Solid medium spotting assay. The growth of *C. auris* wild type and *pmr1*Δ mutant strains were tested by spotting serial dilutions in 10-fold increments onto YPD agar plates supplemented with or without different cell wall-perturbing agents, including Congo Red (CR; 10 μg/ml), CFW (20 μg/ml) or SDS (0.01%). The plates were incubated at 30℃ or at 42℃ as mentioned. Pictures were taken after 2 days. **g** Activation of the cell wall salvage pathway. Crude protein extracts were prepared from exponentially growing cells of *C. auris* wild type and *pmr1*Δ mutant strains and subjected to immunoblot analysis using the antibody that specifically recognizes phosphorylated (activated) Mkc1. Data are expressed as mean ± SD and are

representative of three independent experiments. ****$p < 0.0001$; by two-way ANOVA (**c**). Source data are provided as a Source Data file.

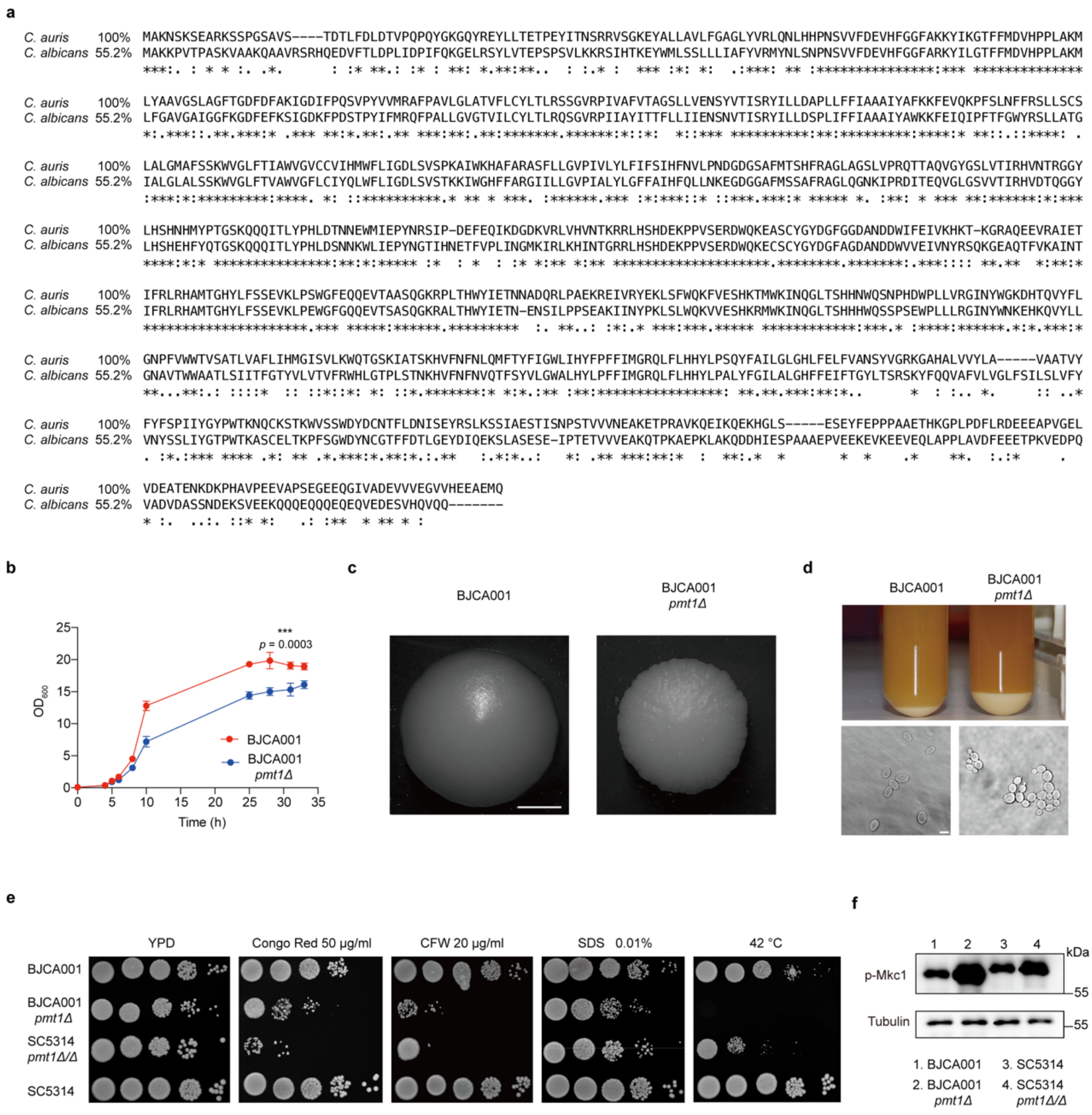

Supplementary Fig.17: The *PMT1* gene is required for cell wall integrity and cell wall composition of *C. auris*. **a** Alignment of deduced amino acid sequences of Pmt1 protein from *C. auris* and *C. albicans*. The homology of deduced protein sequences of Pmt1 from *C. albicans* and *C. auris* was analyzed using the ClustalW alignment tool. **b** Growth curve analysis of *C. auris* WT and *pmr1Δ/Δ* mutant in YPD medium at 30℃ by measuring OD_{600} at the indicated time points (n=3). **c** Representative morphologies of colonies generated from *C. auris* WT or *pmt1Δ/Δ* mutant after 48 h in YPD agar. **d**

C. auris WT and *pmt1Δ/Δ* mutant cells from overnight YPD cultures were washed, diluted, and regrown in fresh YPD medium to logarithmic growth phase at 30℃. Cultures were either photographed (up) or checked for cell morphology by light microscopy (bottom). **e** Solid medium spotting assay. The growth of *C. auris* wild type and *pmt1*∆ mutant strains were tested by spotting serial dilutions in 10-fold increments onto YPD agar plates supplemented with or without different cell wall-perturbing agents, including Congo Red (CR; 50 µg/ml), CFW (20 µg/ml) or SDS (0.01%). The plates were incubated at 30℃ or at 42℃ as mentioned. Pictures were taken after 2 days. **f** Activation of the cell wall salvage pathway. Crude protein extracts were prepared from exponentially growing cells of *C. auris* wild type and *pmt1*∆ mutant strains and subjected to immunoblot analysis using the antibody that specifically recognizes phosphorylated (activated) Mkc1. Data are expressed as mean ± SD and are representative of three independent experiments. ***$p < 0.001$; by two-way ANOVA (**b**). Source data are provided as a Source Data file.

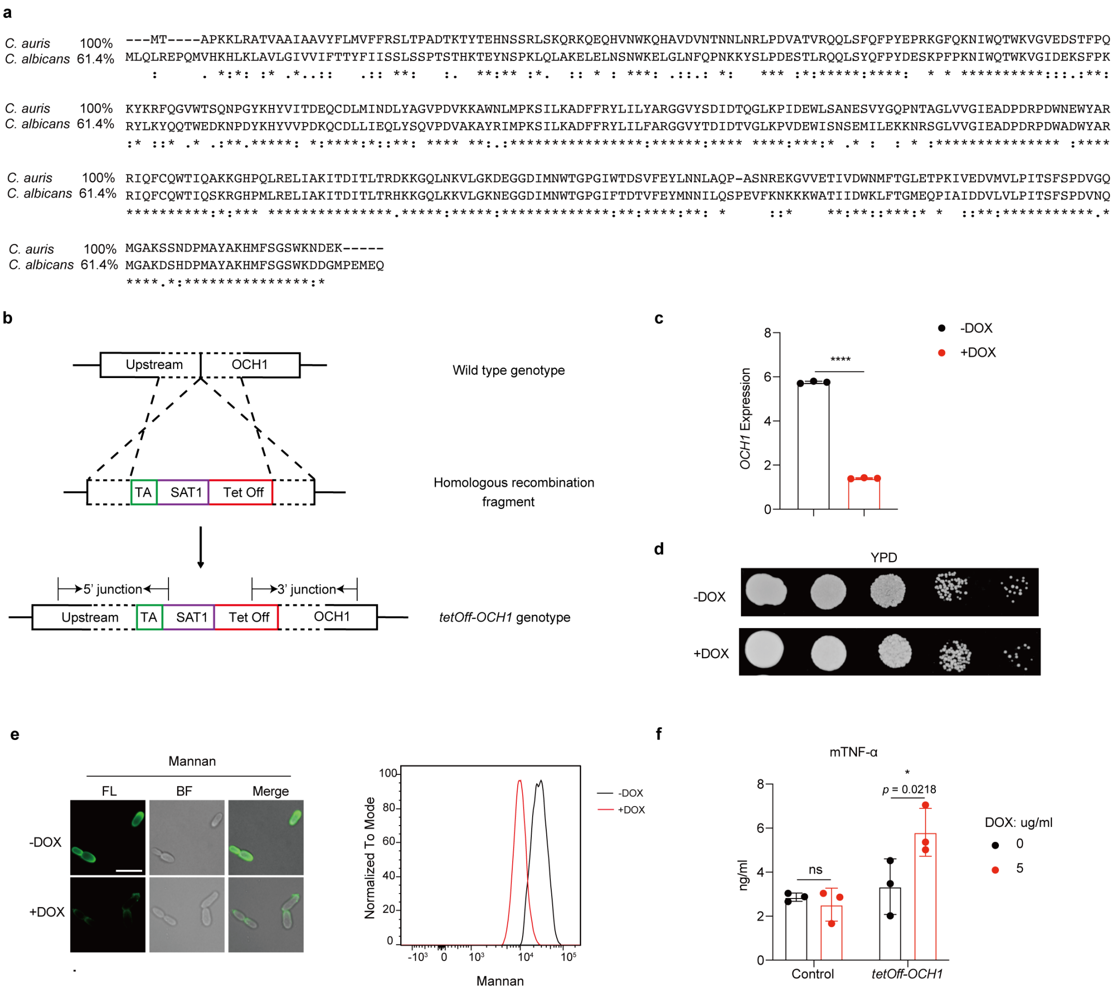

Supplementary Fig.18: Removal of *N*-mannan in *C. auris* effectively induces innate immune activation by β-glucan unmasking. a Alignment of deduced amino acid sequences of Och1 protein from *C. auris* and *C. albicans*. The homology of deduced protein sequences of Och1 from *C. albicans* and *C. auris* was analyzed using the ClustalW alignment tool. **b** Schematic diagram of generation of a conditional Tetoff-*OCH1* mutant in *C. auris*. **c** The regulation of *OCH1* gene expression by doxycycline (DOX; 5 μg/ml) was confirmed by RT-qPCR in the Tetoff-*OCH1* mutant of *C. auris* (n=3). *OCH1* transcript levels were normalized against *ACT1*. **d** Growth curve analysis of *C. auris* WT and TetOff-*OCH1* mutant on YPD supplemented with or without DOX

(up to a concentration of 20 μg/ml). Plates were incubated at 30℃ for 2 days before photography. **e** Representative fluorescence micrographs showing the exposure of *C. auris* cell wall mannan. The Tetoff-*OCH1* mutant strain were inoculated in YPD medium supplemented with or without DOX (5 μg/ml), grown at 30℃ for 6 h (log phase), and then stained with ConA-FITC to visualize mannan. Fluorescence (FL), bright field (BF) and overlay are shown separately. Scale bar, 5 μm. The intensity of fluorescence representing the level of mannan (left) was quantified by flow cytometry (right). **f** BMDMs were co-incubated without (PBS control) or with live TetOff-*OCH1* mutant at a MOI of 5 for 6 h in cell culture medium supplemented with or without DOX (5 μg/ml). The level of TNF-α in the culture supernatants of BMDMs was determined by ELISA (n=3). Data are expressed as mean ± SD and are representative of three independent experiments. *$p < 0.05$; ***$p < 0.001$; by two-side unpaired *t*-test (**c**) or two-way ANOVA with Sidak's test (**f**). Source data are provided as a Source Data file.

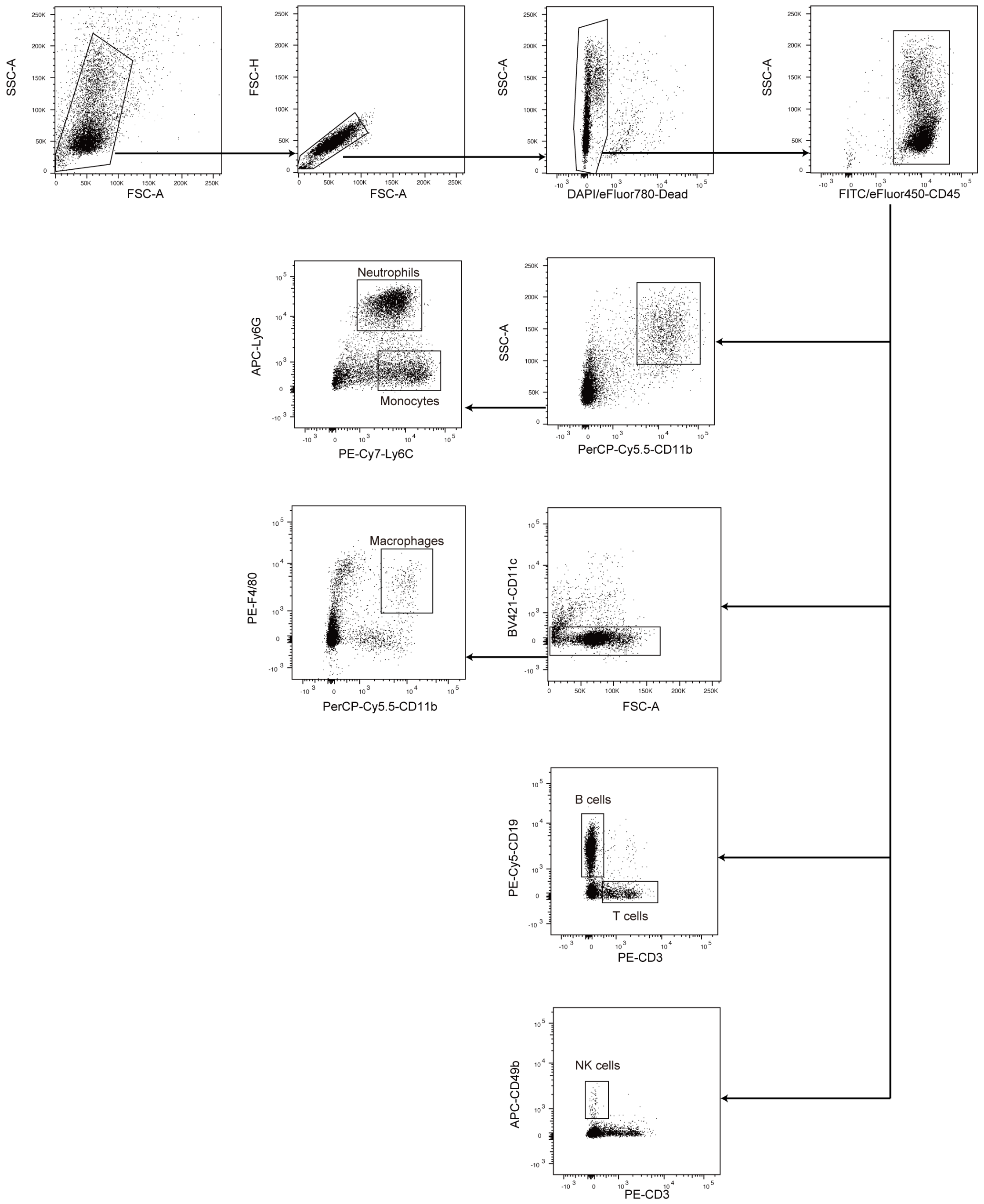

Supplementary Fig.19: Representative Flow gating for the major immune cell population in spleen and kidney of infected mice. All gating was performed off of live cell population. Unstained controls and single color controls were utilized to establish baseline gate settings for each respective antibody–fluorophore(s) combination used in individual experiments.

Supplementary Table 1. Reagents used in this study

Reagents	Source		Identifier
**Chemicals**			
Yeast extract	OXOID		LP0021
Peptone	OXOID		LP0137
CFW	Sigma		F3543
Dulbecco's Modified Eagle's Medium	Gibco		C11995500BT
RPMI-1640	Gibco		22400-089
SYTOX Green	Invitrogen		S7020
Fetal bovine serum	Gibco		10099-141
phenol chloroform	Ambion		AM0883
Trizol	Invitrogen		15596018
**Antibodies**			
Candida β-glucan antibody	1:1000 used	Biosupplies	400-2
Candida Antibody	1:1000 used	Abcam	ab53891
FITC anti-Candida Antibody	1:1000 used	Abcam	ab21164
FC-dectin1a	1:1000 used	Invivogen	fc-hdec1a
Mouse Mincle Detection and Neutralizing antibody - Monoclonal Rat IgG2b	1:200 used	invivogen	mabg-mmcl
Neutralizing monoclonal antibody against mouse Dectin-1	1:200 used	InvivoGen	mabg-mdect
Mouse DC-SIGN/CD209 Antibody	1:500 used	R&D system	MAB83451
Alexa Fluor 568 goat anti-mouse IgG	1:1000 used	Invitrogen	A11004
Alexa Fluor 488 goat anti-human IgG	1:1000 used	Invitrogen	A11013
Alexa Fluor 488 goat anti-mouse IgG	1:1000 used	Invitrogen	A21202
Phospho-SAPK/JNK (Thr183/Tyr185) Rabbit mAb	1:1000 used	Cell Signaling Technology	#4668
SAPK/JNK Antibody	1:1000 used	Cell Signaling Technology	#9252

Phospho-p44/p42 MAPK (Erk1/2 (Thr202/Tyr204) Rabbit mAb	1:1000 used	Cell Signaling Technology	#4370
p44/p42 MAPK (Erk1/2 (Thr202/Tyr204) Rabbit mAb	1:1000 used	Cell Signaling Technology	#4695
Phospho-p38 MAP Kinase (Thr180/Tyr182) Antibody	1:1000 used	Cell Signaling Technology	#9211
p38 MAPK Rabbit mAB	1:1000 used	Cell Signaling Technology	#8690
beta Actin Mouse Monoclonal antibody	1:5000 used	Proteintech	66009-1
CD16/CD32 Monoclonal Antibody (93), eBioscience™	1:200 used	Invitrogen	14-0161-85
Fixable Viability Dye eFluor™ 780	1:1000 used	Invitrogen	65-0865-14
CD45.2 Monoclonal Antibody (104), FITC	1:200 used	Invitrogen	11-0454-82
CD11b Monoclonal Antibody (M1/70), PerCP-Cyanine5.5	1:200 used	Invitrogen	45-0112-80
Ly-6C Monoclonal Antibody (HK1.4), PE-Cyanine7,	1:200 used	Invitrogen	25-5932-82
Ly-6G Monoclonal Antibody (RB6-8C5), APC, eBioscience™	1:200 used	Invitrogen	17-5931-82
CD11c Monoclonal Antibody (N418), BV421	1:200 used	Biolegend	117330
anti-Mouse F4/80 Antibody (T45-2342), PE	1:200 used	BD PharMingen ™	585410
CD45.2 Monoclonal Antibody, eFluor® 450	1:200 used	Invitrogen	48-0454-82
CD19 Monoclonal Antibody (eBio1D3), PE-Cyanine5	1:200 used	Invitrogen	15300470
CD3e Monoclonal Antibody (145-2C11), PE	1:200 used	Invitrogen	12-0031-82
CD49 Monoclonal Antibody (DX5), APC	1:200 used	Invitrogen	17-5971-82
**Critical Commercial Assays**			
PrimeScript RT reagent Kit	Takara		PR047A

TB Green Primer Ex Taq (SYBR Green）	Takara	RR820A
Mouse IL-6 Uncoated ELISA kit	Invitrogen	88-7064
Mouse IL-1 beta Uncoated ELISA	Invitrogen	88-7013
TNF alpha Mouse Uncoated ELISA Kit	Invitrogen	88-7324-77
Mouse CXCL1 ELISA Kit	Proteintech	KE10019
Mouse CXCL2 ELISA Kit	R&D system	DY452
Human IL-1 beta/IL-1F2 DuoSet ELISA	R&D system	DY201
Human IL-6 DuoSet ELISA	R&D system	DY206
Human TNF-alpha DuoSet ELISA	R&D system	DY210
Human CXCL1/GRO alpha DuoSet ELISA	R&D system	DY275
Human CXCL2/GRO beta DuoSet ELISA	R&D system	DY276

Supplementary Table 2. Strains and plasmid used in this study

Strain	Relevant Genotype	Description	Reference
C. albicans **species**			
SC5314	Wild type	Gift from Suzanne M Noble	[1]
mnn4Δ/Δ	*mnn4Δ/Δ*	*mnn4Δ::C.m.LEU2/mnnΔ::C.d. HIS1, his1Δ/his1Δ, arg4Δ/arg4Δ, leu2Δ/leu2Δ, ura3Δ/URA3, iro1Δ/IRO1*	This study
C. auris **species**			
BJCA001	Wild type	China clinical isolate	Gift from Guanghua Huang[7]
caupmr1Δ	*pmr1Δ*	*pmr1:: tetR-NAT-FRT*	This study
caupmt1Δ	*pmt1Δ*	*pmt1:: tetR-NAT-FRT*	This study
TetOff-OCH1	*TetOff-OCH1*	*TA-SAT1-TetOff-OCH1*	This study
BJCA001-R #1	A1	Fluconazole induced	Gift from Guanghua Huang[8]
BJCA001-R #2	B1	Fluconazole induced	Gift from Guanghua Huang[8]
Cau-C #2, Clade I	yCB799	Clinical isolate in China	This study
Cau-C #3, Clade III	BJCA002	Clinical isolate in China	Gift from Guanghua Huang[9]
Cau-C #4, Clade III	RICU2	Clinical isolate in China	Gift from Guanghua Huang[10]
Cau-J #1, Clade II	CBS 10913	Clinical isolate in Japan	Gift from Guanghua Huang [11]
Cau-J #2, Clade II	yCB886	Clinical isolate in Japan	This study
Cau-I #1, Clade I	CBS 12766	Clinical isolate in India	Gift from Guanghua Huang [12]
Cau-I #2, Clade I	CBS 12771	Clinical isolate in India	Gift from LinQi Wang [13]
Cau-I #3, Clade I	CBS 12767	Clinical isolate in India	Gift from LinQi Wang [12]

Cau-I #4, Clade I	CBS 12770	Clinical isolate in India	Gift from LinQi Wang [13]
Cau-I #5, Clade III	CBS 15605	Clinical isolate in India	Gift from Ding Chen [14]
Cau-I #6, Clade III	yCB1033	Clinical isolate in India	This study
Cau-K, Clade II	CBS 12372	Clinical isolate in Korea	Gift from LinQi Wang [9]
Cau-S, Clade IV	CDC B11245	Clinical isolate in South America	Gift from LinQi Wang [15]

**Plasmid**	**Description**	**Source**
pSFS2A	Plasmid with *FLP-SAT1* cassette (generated based on pbluescript)	Johnson lab
pSFS2A-*PMT1*	Plasmid with *FLP-SAT1* cassette,the *PMT1* up flank and *PMT1* down flank	This study
bCB323	Plasmid with TA-SAT1-Tet cassette	This study

Supplementary Table 3. Clinical score of mice to assess severity of disease

Observation	Score points
**I Body weight**	
- No change	0
- Loss of body weight in % = score points; e.g. loss of body weight 8% = 8	1-20
- Loss of body weight ≥20%	20
**II General conditions**	
**Fur**	
- Shining	0
- Matte	2
- Ruffled	4
**Eyes**	
- Clear and clean	0
- Unclean and sticky, closed or semi-closed	3
**Posture**	
- Normal	0
- Hunched	10
- Massively hunched	20
**Clinical complications**	
- Tension, paralysis, tremor	20
- Breath noises	20
- Animal feels cold to the touch	20
**III Motility**	
- Spontaneous (normal behavior, social contacts)	0
- Spontaneous but reduced	1
- Moderately reduced activity	2
- Motility only after stimulation	5
- Isolation, lethargy, coordination disorders	10
- Self-mutilation, aggression	20

Supplementary Table 4. Primers used in this study

Primer Name	Purpose	Sequence
CBO1836	qPCR for mouse *cxcl2*, forward	GCCAAGGGTTGACTTCAAGAAC
CBO1837	qPCR for mouse *cxcl2*, reverse	GCTTCAGGGTCAAGGCAAACT
CBO1850	qPCR for mouse *Tnf-α*, forward	GTCCCCAAAGGGATGAGAAGTT
CBO1851	qPCR for mouse *Tnf-α*, reverse	GTTTGCTACGACGTGGGCTACA
CBO1861	qPCR for mouse *GAPDH*, forward	TGGAGAAACCTGCCAAGTATGA
CBO1862	qPCR for mouse *GAPDH*, reverse	CTGTTGAAGTCGCAGGAGACAA
CBO1863	qPCR for mouse *cxcl1*, forward	CACAGGGGCGCCTATCGCCAA
CBO1864	qPCR for mouse *cxcl1*, reverse	CAAGGCAAGCCTCGCGACCAT
CBO1865	qPCR for mouse *Il-1β*, forward	CAACCAACAAGTGATATTCTCCATG
CBO1866	qPCR for mouse *Il-1β*, reverse	GATCCACACTCTCCAGCTGCA
CBO3085	common AgNAT, forward	ATCAAGCTTGCCTCGTCCCCGCC
CBO3086	common AgNAT, reverse	TTAGTATCGAATCGACAGCAGTATAG
CBO3088	upstream fragment for *PMR1* delete, reverse	GGCGGGGACGAGGCAAGCTTGATTCGTGAGTCAACTCGTTATCGCCATGGATC
CBO3089	downstream fragment for *PMR1* delete, forward	GCTATACTGCTGTCGATTCGATACTAAACTGAAGCCTTGTCATTCACAGAC
CBO3103	*PMR1* ORF check, forward	CAGGCGTTCAACGACCCGTTGATC
CBO3104	*PMR1* ORF check, reverse	GCGTACCAATGTGCCCATGAAAG
CBO3120	upstream fragment for *PMR1* delete, forward	GATCTCCGTTAAATTGTGGCTAAG

CBO3121	downstream fragment for *PMR1* delete, reverse	GCTGATCTTTTTAACCCAGACACT
CBO3137	qPCR for *OCH1* ORF, forward	AAGACGTACACTGAGCACAATAGC
CBO3138	qPCR for *OCH1* ORF, reverse	ACATCGGGAAGTCTGTTGAGAT
CBO3139	qPCR for *PMR1* ORF, forward	TTGTCACTGTTACACTAGCCTTGG
CBO3140	qPCR for *PMR1* ORF, reverse	CTGGGTCAAGGTACCAGTCTTATC
CBO3168	*C. auris PMT1* 5' flank, forward	GCGAATTGGGTACCGGGCCCAGTCCCGCATGAACCACTTACG
CBO3169	*C. auris PMT1* 5' flank, reverse	AGTATAGGAACTTCCTCGAGTGTACTCTGGGGTTTCGGTCAACAAGTAC
CBO3170	*C. auris PMT1* 3' flank, forward	ACTAGTTCTAGAGCGGCCGCACAAGCCTCATGCCGTTC
CBO3171	*C. auris PMT1* 3' flank, reverse	AAGCTGGAGCTCCACCGCGGCTAGTAGCTCCAACAATTTGGATCTCG
CBO3173	*C. albicans TetOff-OCH1 3*' flank, reverse	CTGGCCATACACAGACTCATTG
CBO3220	*PMR1* upstream verification primer, forward	GCACAGGGAACAGAAAGAATAGTT
CBO3221	*PMR1* upstream verification primer, reverse	CAAGACTGTCAAGGAGGGTATTCT
CBO3222	*PMR1* downstream verification primer, forward	GTATGTGAATGCTGGTCGCTATAC
CBO3223	*PMR1* downstream verification primer, reverse	ATCTCCAGTGATAGATTGCAGATG
CBO3253	verification primer for *PMT1* upstream fragment in pSFS2A and junction in *C. auris*, reverse	ACTCAGAGCACGCTAGACAA
CBO3254	verification primer for *PMT1* downstream fragment in pSFS2A, forward	GCGCACAGTCTCATCGAATT
CBO3266	verification primer for *PMT1* upstream fragment in pSFS2A and upstream junction in *C. auris*, forward	ATGTGCTGCAAGGCGATTA

CBO3306	verification primer for *PMT1* downstream fragment in pSFS2A, reverse	TATGTTGTGTGGAATTGTGAGC
CBO3341	*C. auris PMT1* ORF PCR, forward	TTAAGAAGTTCGAAGTGCAGAAAC
CBO3342	*C. auris PMT1* ORF PCR, reverse	AGATCTGTTGTAAGGCTCAATCAT
CBO3343	*C. albicans TetOff-OCH1* 5' flank, forward	TAAGACGGGGTTGCCTGTCTTTT
CBO3345	qPCR for *C. auris PMT1* ORF, forward	GGAAGAGCCCAGGAAGAAGT
CBO3346	qPCR for *C. auris PMT1* ORF, reverse	GACTTCCTGCTGCTCGAAAC
CBO3347	*C. auris* verification primer for *PMT1* upstream junction, forward	CACGCTCGATTGCTGACATG
CBO3348	*C. auris* verification primer for *PMT1* downstream junction, reverse	TGCTCCTCATTCTGCCTCTC
CBO3286	Common 5' verification primer for Leu 5' flank, reverse	AGAATTCCCAACTTTGTCTG
CBO3287	Common 3' verification primer for Leu 3' flank, forward	AAACTTTGAACCCGGCTGCG
CBO3288	Common 5' verification primer for His 5' flank, reverse	ATTAGATACGTTGGTGGTTC
CBO3289	Common 3' verification primer for His 3' flank, forward	AACACAACTGCACAATCTGG
CBO4436	common *TetOff*, forward	cgaaaagtgccacctgacgtctaa
CBO4472	*C. albicans TetOff-OCH1* 5' flank, reverse	ttagacgtcaggtggcacttttcgAAAAGCGAGTTTAAGTGGTGG
CBO4473	common *TetOff*, reverse	cggtatgaccatgattacct
CBO4474	*C. albicans TetOff-OCH1 3*' flank, forward	aggtaatcatggtcataccgATGACGGCCCCGAAGAAGCTTA
CBO4475	*C. albicans MNN4* 5' flank, forward	CCAACACATCCAGGCAAATA
CBO4476	*C. albicans MNN4* 5' flank, reverse	CACGGCGCGCCTAGCAGCGGGGTTGGAGTGTTGTATGTATATG
CBO4477	*C. albicans MNN4* 3' flank, forward	GTCAGCGGCCGCATCCCTGCCTGGTAGTTCTGATAATGGG

CBO4478	*C. albicans MNN4* 3' flank, reverse	CACGTGGTCAAAGAACGAAA
CBO4479	*C. albicans MNN4* upstream verification primer, forward	GAAGTTCACGAATACAACATC
CBO4480	*C. albicans MNN4* downstream verification primer, reverse	TATTGAGGTGTCTCTGAAG
CBO4481	*C. albicans MNN4* orf PCR, forward	TGAGCAATCGTCAAAACCAG
CBO4482	*C. albicans MNN4* orf PCR, reverse	GGTTTCCATCATTGGCTTTC

References

1. Fonzi, W.A. & Irwin, M.Y. Isogenic strain construction and gene mapping in Candida albicans. *Genetics* **134**, 717-28 (1993).
2. Huang, G. *et al.* Bistable expression of WOR1, a master regulator of white-opaque switching in Candida albicans. *Proc Natl Acad Sci U S A* **103**, 12813-8 (2006).
3. Huang, G., Srikantha, T., Sahni, N., Yi, S. & Soll, D.R. CO(2) regulates white-to-opaque switching in Candida albicans. *Curr Biol* **19**, 330-4 (2009).
4. Noble, S.M., French, S., Kohn, L.A., Chen, V. & Johnson, A.D. Systematic screens of a Candida albicans homozygous deletion library decouple morphogenetic switching and pathogenicity. *Nat Genet* **42**, 590-8 (2010).
5. Liao, R.S., Rennie, R.P. & Talbot, J.A. Novel fluorescent broth microdilution method for fluconazole susceptibility testing of Candida albicans. *J Clin Microbiol* **39**, 2708-12 (2001).
6. Tao, L. *et al.* Discovery of a "white-gray-opaque" tristable phenotypic switching system in candida albicans: roles of non-genetic diversity in host adaptation. *PLoS Biol* **12**, e1001830 (2014).
7. Wang, X. *et al.* The first isolate of Candida auris in China: clinical and biological aspects. *Emerg Microbes Infect* **7**, 93 (2018).
8. Bing, J. *et al.* Experimental evolution identifies adaptive aneuploidy as a mechanism of fluconazole resistance in Candida auris. *Antimicrob Agents Chemother* (2020).
9. Fan, S. *et al.* A biological and genomic comparison of a drug-resistant and a drug-susceptible strain of Candida auris isolated from Beijing, China. *Virulence* **12**, 1388-1399 (2021).
10. Tian, S. *et al.* First cases and risk factors of super yeast Candida auris infection or colonization from Shenyang, China. *Emerg Microbes Infect* **7**, 128 (2018).
11. Satoh, K. *et al.* Candida auris sp. nov., a novel ascomycetous yeast isolated from the external ear canal of an inpatient in a Japanese hospital. *Microbiol Immunol* **53**, 41-4 (2009).
12. Bing, J. *et al.* A case of Candida auris candidemia in Xiamen, China, and a comparative analysis of clinical isolates in China. *Mycology* **13**, 68-75 (2022).
13. Vatanshenassan, M. *et al.* Evaluation of Microsatellite Typing, ITS Sequencing, AFLP Fingerprinting, MALDI-TOF MS, and Fourier-Transform Infrared Spectroscopy Analysis of Candida auris. *J Fungi (Basel)* **6**(2020).
14. Hernando-Ortiz, A. *et al.* Virulence of Candida auris from different clinical origins in Caenorhabditis elegans and Galleria mellonella host models. *Virulence* **12**, 1063-1075 (2021).

15. Bentz, M.L., Sexton, D.J., Welsh, R.M. & Litvintseva, A.P. Phenotypic switching in newly emerged multidrug-resistant pathogen Candida auris. *Med Mycol* (2018).
