# Supplementary material for: Innate immune responses against the fungal pathogen Candida auris
Source: Nat Commun. 2022 Jun 21;13:3553. doi: 10.1038/s41467-022-31201-x (PMC9213489; doi:10.1038/s41467-022-31201-x)
Supplement: Supplementary file 2 — Reporting Summary [file 41467_2022_31201_MOESM2_ESM.pdf]

nature research

Corresponding author(s): Changbin Chen, Dongsheng Zhou

Last updated by author(s): May 25, 2022

Reporting Summary

Nature Research wishes to improve the reproducibility of the work that we publish. This form provides structure for consistency and transparency in reporting. For further information on Nature Research policies, see our Editorial Policies and the Editorial Policy Checklist.

Statistics

For all statistical analyses, confirm that the following items are present in the figure legend, table legend, main text, or Methods section.

n/a	Confirmed	
☐	☒	The exact sample size (*n*) for each experimental group/condition, given as a discrete number and unit of measurement
☐	☒	A statement on whether measurements were taken from distinct samples or whether the same sample was measured repeatedly
☐	☒	The statistical test(s) used AND whether they are one- or two-sided<br>*Only common tests should be described solely by name; describe more complex techniques in the Methods section.*
☒	☐	A description of all covariates tested
☐	☒	A description of any assumptions or corrections, such as tests of normality and adjustment for multiple comparisons
☐	☒	A full description of the statistical parameters including central tendency (e.g. means) or other basic estimates (e.g. regression coefficient) AND variation (e.g. standard deviation) or associated estimates of uncertainty (e.g. confidence intervals)
☐	☒	For null hypothesis testing, the test statistic (e.g. *F*, *t*, *r*) with confidence intervals, effect sizes, degrees of freedom and *P* value noted<br>*Give P values as exact values whenever suitable.*
☒	☐	For Bayesian analysis, information on the choice of priors and Markov chain Monte Carlo settings
☐	☒	For hierarchical and complex designs, identification of the appropriate level for tests and full reporting of outcomes
☒	☐	Estimates of effect sizes (e.g. Cohen's *d*, Pearson's *r*), indicating how they were calculated

Our web collection on statistics for biologists contains articles on many of the points above.

Software and code

Policy information about availability of computer code

Data collection	BD FACSDiva Software (V6.0) was used to collect all flow cytometry data.
Data analysis	Prism 8 (V8.2.1) for statistical analysis; ImageJ (V2.3.0) for image analysis;Skewer (V0.2.2), STAR (V2.7.4), StringTie (V2.1.2) and DESeq2 (V1.20) for RNA-seq analysis; Flowjo 10.4 for flow cytometry data analysis;Adobe PhotoShop CC (V20.0.7) for Western blot and micrograph images.

For manuscripts utilizing custom algorithms or software that are central to the research but not yet described in published literature, software must be made available to editors and reviewers. We strongly encourage code deposition in a community repository (e.g. GitHub). See the Nature Research guidelines for submitting code & software for further information.

Data

Policy information about availability of data

All manuscripts must include a data availability statement. This statement should provide the following information, where applicable:

- Accession codes, unique identifiers, or web links for publicly available datasets
- A list of figures that have associated raw data
- A description of any restrictions on data availability

The authors declare that the data supporting the findings of this study are available within the article and its Supplementary Information files. RNASeq data can be found under the GEO accession number GSE203508（https://www.ncbi.nlm.nih.gov/geo/).

Field-specific reporting

Please select the one below that is the best fit for your research. If you are not sure, read the appropriate sections before making your selection.

☒ Life sciences ☐ Behavioural & social sciences ☐ Ecological, evolutionary & environmental sciences

For a reference copy of the document with all sections, see nature.com/documents/nr-reporting-summary-flat.pdf

Life sciences study design

All studies must disclose on these points even when the disclosure is negative.

Sample size	No statistical methods were used to predetermine sample size. Sample sizes were estimated based on standards of the field and preliminary experiments. For in vitro experiment, 3 - 6 sample size was used for analysis; For in vivo experiment , 4-10 mice per group were used. These sample sizes were sufficient to detect meaningful biological difference with good reproducibility.
Data exclusions	No data were excluded from the analyses.
Replication	All results were repeated at least three independent times unless otherwise specified. Similar data were obtained in independent experiments.
Randomization	For the animal experiments, mice were randomly allocated to different experimental groups.
Blinding	To reduce potential experimental bias, the investigators were blinded to group allocation during data collection and analysis for H&E staining and PAS staining. While blinding to group allocation was not always posssible due to COVID19 restrictions, the data collection and analysis were performed carefully by two different investigators.

Reporting for specific materials, systems and methods

We require information from authors about some types of materials, experimental systems and methods used in many studies. Here, indicate whether each material, system or method listed is relevant to your study. If you are not sure if a list item applies to your research, read the appropriate section before selecting a response.

Materials & experimental systems

n/a	Involved in the study	
☐	☒	Antibodies
☐	☒	Eukaryotic cell lines
☒	☐	Palaeontology and archaeology
☐	☒	Animals and other organisms
☐	☒	Human research participants
☒	☐	Clinical data
☒	☐	Dual use research of concern

Methods

n/a	Involved in the study	
☒	☐	ChIP-seq
☐	☒	Flow cytometry
☒	☐	MRI-based neuroimaging

Antibodies

Antibodies used	All antibody informations were provided in Supplementary Table 1.
Validation	All antibodies used in this study are commercial and used according to the manufacturer's instructions.

Eukaryotic cell lines

Policy information about cell lines

Cell line source(s)	All the cell lines used are from ATCC. L929 cells (ATCC® CRL-6364); HaCat cells ; Caco-2 cells (ATCC® HTB-37); HUVEC cells (ATCC® CRL-1730); A549 cells (ATCC® CRM-CCL-185); Hela cells (ATCC® CRM-CCL-2)
Authentication	All cell lines were not authorized.
Mycoplasma contamination	All cell lines tested negative of mycoplasma contamination.
Commonly misidentified lines (See ICLAC register)	No commonly misidentified cell lines are used.

Animals and other organisms

Policy information about studies involving animals; ARRIVE guidelines recommended for reporting animal research

Laboratory animals	Female C57BL/6 mice (6-8 weeks old, weighing 18-20g) were purchased from Vital River Laboratory Animal Technology Company. The mice were routinely maintained in a pathogen-free animal facility at a temperature of 21°C, relative humidity of 50-70% and under a constant 12-h light/dark cycle. Mice were given free access to food and water throughout the study. All the procedures were conducted in compliance with a protocol approved by the Institutional Animal Care and Use Committee (IACUC) at Institut Pasteur of Shanghai, CAS.
Wild animals	The study did not involve wild animals.
Field-collected samples	The study did not involve samples collected from the field.
Ethics oversight	All animal experiments were performed in compliance with the Regulations for the Care and Use of Laboratory Animals issued by the Ministry of Science and Technology of the People's Republic of China, which enforces the ethical use of animals. The protocol was approved by IACUC at the Institut Pasteur of Shanghai, Chinese Academy of Sciences (Permit Number: A2020016).

Note that full information on the approval of the study protocol must also be provided in the manuscript.

Human research participants

Policy information about studies involving human research participants

Population characteristics	Human research participants who were voluntary to donor blood.
Recruitment	Not specific patients were recruited for this study. All human blood was collected in Shanghai General Hospital, Shanghai Jiao Tong University School of Medicine and provided as unselected samples to ensure blinding approach.
Ethics oversight	Human blood was obtained from volunteering donors with oral consent through a protocol that was approved by the Institutional Review Board and Human Ethics Committee of Shanghai General Hospital, Shanghai Jiao Tong University School of Medicine.

Note that full information on the approval of the study protocol must also be provided in the manuscript.

Flow Cytometry

Plots

Confirm that:

- [ ] The axis labels state the marker and fluorochrome used (e.g. CD4-FITC).
- [x] The axis scales are clearly visible. Include numbers along axes only for bottom left plot of group (a 'group' is an analysis of identical markers).
- [x] All plots are contour plots with outliers or pseudocolor plots.
- [x] A numerical value for number of cells or percentage (with statistics) is provided.

Methodology

Sample preparation	Spleens were removed of any connective tissues and mechanically digested in cold FACS buffer to produce a single cell suspension. After passing through a 100-µm filter, the remaining red cells were lysed with RBC lysing buffer. Red cell-free cell suspensions were centrifuged at 400 g for 10 min at 4˚C and splenocytes were suspended in 8 ml of FACS buffer after passing through a 40-µm filter. In similar, kidneys were decapsulated and finely chopped with surgical scissors before enzymatic digestion at 37̊C in 3 ml of 1% DMEM containing 0.5 mg/ml collagenase and 0.2 mg/ml DNase I (Roche) for 45 min with intermittent shaking. Mechanical dissociation with an 18-gauge needle resulted in a single cell suspension and the digested tissue was passed through a 70-µm filter, washed, and the remaining red cells were lysed with ACK lysis buffer. Red cell-free cell suspensions were then treated from the same preparations as above. Leukocyte enrichment from kidneys suspended in 40% Percoll (GE Healthcare) was performed by overlay of the cell suspensions on 70% Percoll, and centrifuged at 600 g for 20 min at RT. The leukocytes enriched at the interphase were isolated, washed three times in 5% DMEM and suspended in FACS buffer. A total of 1 × 106 live cells were blocked with CD16/CD32 and stained with antibodies listed in Supplementary Table 1.
Instrument	BD Fortessa
Software	BD FACSDiva for data acquisition and FlowJo 10.4 for post-acquisition analyzes.
Cell population abundance	No FACS sorting was performed in this study.
Gating strategy	Cell debris were excluded by SSC-A and FSC-A gating (G1), Doublets were excluded by FSC-A and FSC-H gating (G2) and SSC-A and Fixable Viability Dye positive(G3) for all flow cytometry analysis. Leukocytes cells were gated by CD45 positive (G4); CD11b+ cells were gated by CD11b positive (G5), Neutrophils were gated by Ly6G high and Ly6C low (G6); Monocytes were gated by Ly6C high (G7); Macrophages were gated by CD11c negative, CD11b positive and F4/80 positive (G8). NK cells were gated by CD49 positive(G9); B cells were gated by CD19 positive (G10) and T cells were gated by CD3 positive (G11). A

detailed description of gating strategy is provided in Supplementary Fig. 19.

☒ Tick this box to confirm that a figure exemplifying the gating strategy is provided in the Supplementary Information.
