# Supplementary material for: Innate immune responses against the fungal pathogen Candida auris
Source: Nat Commun. 2022 Jun 21;13:3553. doi: 10.1038/s41467-022-31201-x (PMC9213489; doi:10.1038/s41467-022-31201-x)
Supplement: Supplementary file 27 — Description of Additional Supplementary Files [file 41467_2022_31201_MOESM27_ESM.pdf]

Title: Supplementary Data 1

Description: Differentially expressed genes (DEGs) in BMDMs treated with live *C. auris* or *C. albicans* yeast cells (MOI=5; 3 h).

The *p*-value was calculated by the Wald test within DESeq2 and adjusted using Benjamini-Hochberg correction for multiple comparisons.

Title: Supplementary Data 2

Description: Differentially expressed genes (DEGs) in BMDMs treated with live *C. auris* or *C. albicans* yeast cells (MOI=5; 6 h).

The *p*-value was calculated by the Wald test within DESeq2 and adjusted using Benjamini-Hochberg correction for multiple comparisons.

Title: Supplementary Data 3

Description: Significantly enriched KEGG pathways of DEGs in BMDMs treated with live *C. auris* or *C. albicans* yeast cells (MOI=5; 3 h).

The *p*-value was calculated based on the cumulative hypergeometric distribution and adjusted using Benjamini-Hochberg correction for multiple comparisons.

Title: Supplementary Data 4

Description: Significantly enriched KEGG pathways of DEGs in BMDMs treated with live *C. auris* or *C. albicans* yeast cells (MOI=5; 6 h).

The *p*-value was calculated based on the cumulative hypergeometric distribution and adjusted using Benjamini-Hochberg correction for multiple comparisons.

Title: Supplementary Data 5

Description: A list of the putative mannosyltransferases-encoding genes in *C. auris* and *C. albicans.*

Title: Supplementary Movie 1

Description: Co-incubation of *C. auris* with Caco-2 cell line. Total time is 5 hours from the start to the end of the recording (20 frames/sec, 1 sec in video equals 20min in real time). Images from this video are displayed in Supplementary Fig.5.

Title: Supplementary Movie 2

Description: Co-incubation of *C. albicans* with Caco-2 cell line. Total time is 5 hours from the start to the end of the recording (20 frames/sec, 1 sec in video equals 20min in real time). Images from this video are displayed in Supplementary Fig.5.

Title: Supplementary Movie 3

Description: Co-incubation of *C. auris* with Hacat cell line. Total time is 5 hours from

the start to the end of the recording (20 frames/sec, 1 sec in video equals 20min in real time).

Title: Supplementary Movie 4

Description: Co-incubation of *C. albicans* with Hacat cell line. Total time is 5 hours from the start to the end of the recording (20 frames/sec, 1 sec in video equals 20min in real time).

Title: Supplementary Movie 5

Description: Co-incubation of *C. auris* with HUVEC cell line. Total time is 5 hours from the start to the end of the recording (20 frames/sec, 1 sec in video equals 20min in real time).

Title: Supplementary Movie 6

Description: Co-incubation of *C. albicans* with HUVEC cell line. Total time is 5 hours from the start to the end of the recording (20 frames/sec, 1 sec in video equals 20min in real time).

Title: Supplementary Movie 7

Description: Co-incubation of *C. auris* with A549 cell line. Total time is 5 hours from the start to the end of the recording (20 frames/sec, 1 sec in video equals 20min in real time).

Title: Supplementary Movie 8

Description: Co-incubation of *C. albicans* with A549 cell line. Total time is 5 hours from the start to the end of the recording (20 frames/sec, 1 sec in video equals 20min in real time).

Title: Supplementary Movie 9

Description: Co-incubation of *C. auris* with Hela cell line. Total time is 5 hours from the start to the end of the recording (20 frames/sec, 1 sec in video equals 20min in real time).

Title: Supplementary Movie 10

Description: Co-incubation of *C. albicans* with Hela cell line. Total time is 5 hours from the start to the end of the recording (20 frames/sec, 1 sec in video equals 20min in real time).

Title: Supplementary Movie 11

Description: Co-incubation of *C. auris* with mouse BMDMs. Total time is 1 hour from the start to the end of the recording (5 frames/sec, 1 sec in video equals 5min in real time). Images from this video are displayed in Fig.2.

Title: Supplementary Movie 12

Description: Co-incubation of *C. albicans* with mouse BMDMs. Total time is 1 hours from the start to the end of the recording (5 frames/sec, 1 sec in video equals 5min in real time). Images from this video are displayed in Fig.2.

Title: Supplementary Movie 13

Description: Co-incubation of *C. auris* with human MDMs. Total time is 1 hour from the start to the end of the recording (5 frames/sec, 1 sec in video equals 5min in real time). Images from this video are displayed in Supplementary Fig.6.

Title: Supplementary Movie 14

Description: Co-incubation of *C. albicans* with human MDMs. Total time is 1 hour from the start to the end of the recording (5 frames/sec, 1 sec in video equals 5min in real time). Images from this video are displayed in Supplementary Fig.6.

Title: Supplementary Movie 15

Description: Co-incubation of *C. auris* with mouse neutrophils. Total time is 1 hour from the start to the end of the recording (5 frames/sec, 1 sec in video equals 5min in real time). Images from this video are displayed in Fig.2.

Title: Supplementary Movie 16

Description: Co-incubation of *C. albicans* with mouse neutrophils. Total time is 1 hour from the start to the end of the recording (5 frames/sec, 1 sec in video equals 5min in real time). Images from this video are displayed in Fig.2.

Title: Supplementary Movie 17

Description: Co-incubation of *C. auris* with human neutrophils. Total time is 1 hour from the start to the end of the recording (25 frames/sec, 1 sec in video equals 5min in real time). Images from this video are displayed in Supplementary Fig.6.

Title: Supplementary Movie 18

Description: Co-incubation of *C. albicans* with human neutrophils. Total time is 1 hour from the start to the end of the recording (5 frames/sec, 1 sec in video equals 5min in real time). Images from this video are displayed in Supplementary Fig.6.
